# Probing sulfotransferase binding and inhibition with synthetic PAPS analogs reveals the role of the 3′-phosphate and informs molecular tool design

**DOI:** 10.1039/d6cb00094k

**Published:** 2026-06-19

**Authors:** Maria Matveeva, Agnieszka Młynarska-Cieślak, Mikołaj Chromiński, Tomasz Spiewla, Jonathan W. Mueller, Jacek Jemielity, Joanna Kowalska

**Affiliations:** a Division of Biophysics, Institute of Experimental Physics, Faculty of Physics, University of Warsaw, Pasteura 5, 02-093 Warsaw Poland jkowalska@fuw.edu.pl; b Centre of New Technologies University of Warsaw, Banacha 2c, 02-097 Warsaw Poland; c Department of Metabolism and Systems Science, School of Medical Sciences, College of Medicine and Health, University of Birmingham Birmingham UK

## Abstract

Sulfotransferases (STs) are a class of enzymes that catalyze the transfer of the sulfuryl group (–SO_3_^−^), most commonly from the universal cofactor 3′-phosphoadenosine 5′-phosphosulfate (PAPS), to nucleophilic acceptors bearing hydroxyl or amino groups. Although this process is widespread in biology, it involves the energy-intensive synthesis of PAPS and the generation of a natural inhibitor of sulfotransferases – the bis-phosphorylated nucleotide 3′-phosphoadenosine 5′-phosphate (PAP). This study aims to provide a deeper insight into the structure–activity relationship of the PAPS–ST interaction and lay the groundwork for PAPS-derived molecular tool design. To this end, we report a library of chemically modified PAPS and PAP analogs and evaluate their binding and inhibitory properties toward the plant sulfotransferase AtSOT18 and mammalian SULT1A3. Using microscale thermophoresis, it was found that STs do not differentiate between the 5′-phosphosulfate and 5′-diphosphate analogs, making the latter non-functional mimics potentially useful for structural studies and revealing an important role of the 3′-phosphate in ligand recognition and binding specificity. The modifications at the 2′-, 5′-sites, or at the N6 and C8 positions of the adenine rarely made significant contributions to the stabilization of the complex. A strong preference for the adenine base over alternative nitrogenous bases in the cofactor structure was, however, observed. The use of ^19^F NMR spectroscopy as a molecular tool for screening potential inhibitors of AtSOT18 and SULT1A3 highlighted C8-(1-amino-2-azidoethyl)-substituted analogs as promising compounds for targeting STs. Overall, the study revealed several novel aspects of molecular recognition of PAPS by STs and laid the groundwork for future molecular tool design.

## Abstract 2

Screening of 16 synthetic PAP/PAPS analogs by MST and ^19^F NMR identified the 3′-phosphate as a major determinant of sulfotransferase recognition, while revealing broad tolerance toward other cofactor modifications.

## Graphical abstract

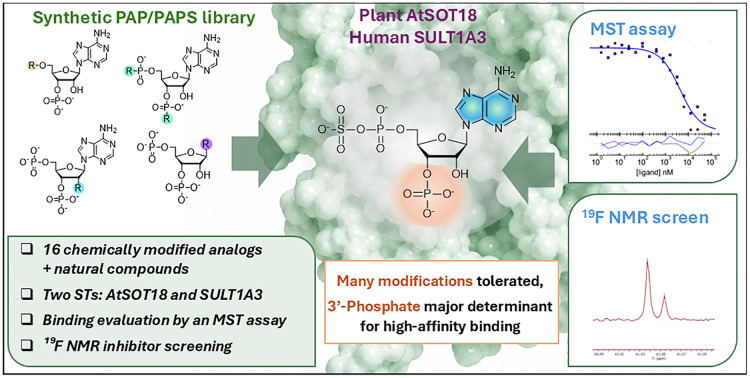

## Introduction

Sulfotransferases (STs) are enzymes that catalyze sulfuryl group (–SO_3_^−^) transfer in diverse biological processes.^1^ These enzymes have been found in metabolically active tissues such as the liver, lung, brain, platelets, kidney, and small intestine. High expression levels in these tissues indicate a key role of sulfation in the detoxification and metabolic activation of small biomolecules, including xenobiotics, neurotransmitters, and hormones, demonstrating the direct involvement in hormone regulation, molecular recognition, and cellular signaling.^2^ Moreover, the fact that low levels of ST expression correlate with cancer^3,4^ and neurological diseases^5,6^ has led to the search for ST inhibitors, activity probes, and other dedicated molecular tools to understand their role in these processes better.

**Fig. 1:**
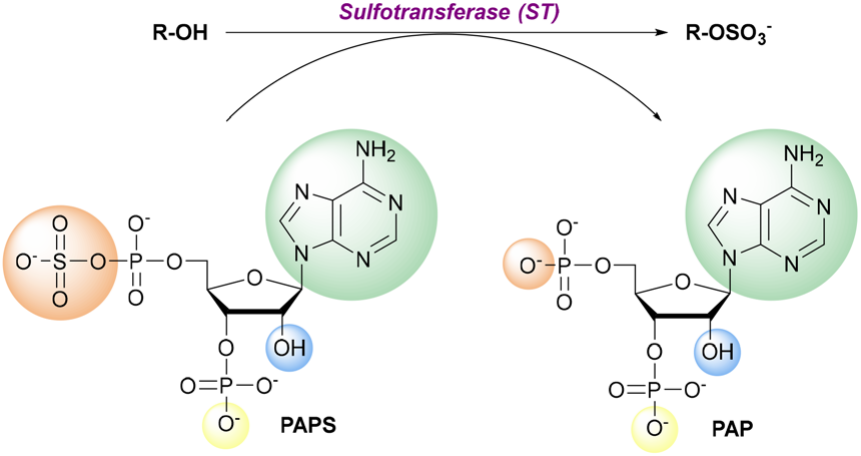
General catalytic activity of sulfotransferases. Potential modification sites are marked with colors in the PAPS/PAP structure.

3′-Phosphoadenosine 5′-phosphosulfate (PAPS) is used by most STs as a universal sulfuryl moiety donor, facilitating its transfer to various acceptor molecules and releasing 3′-phosphoadenosine 5′-phosphate (PAP) as a byproduct (Fig. 1).^7,8^ Various strategies for studying and inhibiting sulfotransferases have been explored: using small molecules,^9–11^ PAPS-derived inhibitors,^12–14^ or bisubstrate analogs.^15–17^ Small-molecule substrate analogs of sulfotransferases often potently inhibit sulfotransferases, but their applicability is usually limited to a single specific enzyme, which is not suitable for many advanced applications.

Among analytical methods for studying STs, the use of radioactive labeled [^35^S]PAPS^13,18^ dominates, followed by fluorescence-based methods, which utilize a fluorescent label present in the substrate or its analog,^10,11,19–27^ the enzyme itself,^28^ or co-products released during the reaction.^29^ Mass spectrometry is increasingly employed for both quantitative and qualitative analysis of protein–ligand interactions without the need for labeling, allowing the determination of ST levels in individual tissues.^30^

However, despite these significant developments in ST-related research, molecular tools based on the universal cofactor (PAPS) structure have rarely been developed or studied.^31–34^ We envisioned that, by analogy to kinases, such tools could open up enormous possibilities, such as the transfer of reactive or fluorescent moieties, covalent crosslinking, or the development of biorthogonal sulfuryl-transfer systems with applications in proteomics, *in cellulo* protein tagging or targeting, and many other areas.

**Fig. 2:**
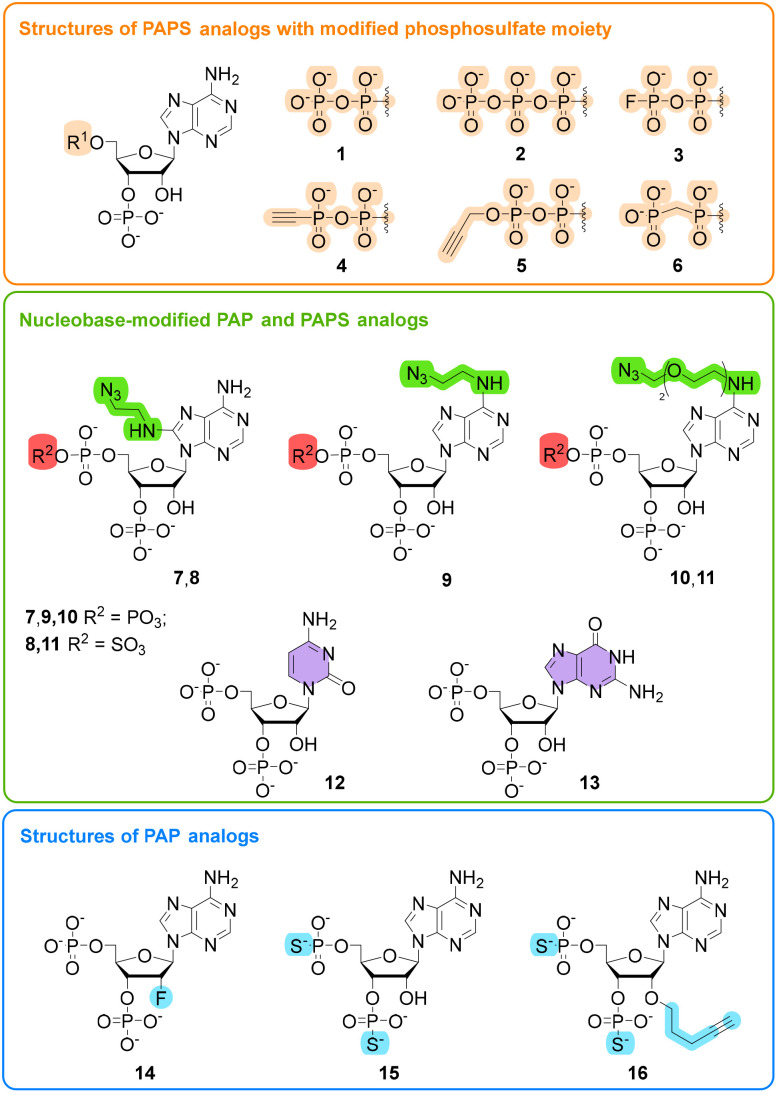
Structures of the compounds synthesized in this study 1–16.

As a first step in this direction, in this work, we created a library of 1–16 chemically modified PAPS and PAP analogs and evaluated their ability to bind and inhibit model STs (Fig. 2). The results gave us unprecedented insight into the structure–activity relationship for PAPS–ST interaction and allowed us to identify the most promising modification sites and types. Unexpectedly, the results of our study also shed new light on the role of the 3′-phosphate moiety present in the PAPS structure.

## Results and discussion

### Key features of PAPS analog collection

The compounds synthesized in this study to evaluate the structure–activity relationship of the PAPS–ST interaction are shown in Fig. 2. We divided them into several groups, based on the modification site. The first group contains compounds 1–6 modified at the 5′-site by replacing the 5′-phosphosulfate moiety with another group, such as diphosphate, fluorophosphate, bisphosphonate, and others. The second group includes compounds modified with the nucleobase moiety, either by functionalizing the adenosine with clickable linkers 7–11 or replacing the whole adenine moiety with cytosine (12) or guanine (13). The third group includes three compounds modified with the 5′,3′-diphosphoribose moiety, by functionalizing the 2′-position of the ribose or introducing phosphorothioate moieties 14–16.

From a functional perspective, these compounds can act either as functional PAPS analogs (ST substrates) or potential inhibitors. As potential functional analogs, we considered compounds containing the unmodified 5′-phosphosulfate group and modifications at sites not directly involved in catalysis. These sites can accommodate the introduction of various functional linkers enabling tagging or labeling at a later stage to facilitate ST activity monitoring. In contrast, all analogs in which the phosphosulfate group was truncated or replaced by another moiety were expected to act as sulfotransferase binders (and, potentially, inhibitors) that could be harnessed as molecular tools for structural and functional studies. Relevant modifications in 1–6 included the introduction of a phosphate (same volume and larger charge), fluorophosphate group (same volume, same charge), C-phosphonate or propargyl phosphate groups (allowing for labeling), or a methylene(bisphosphonate) moiety (conferring hydrolytic stability).

The adenine moiety of PAPS interacts with amino acids in the ST binding pocket and stabilizes PAPS in the complex prior to sulfate group transfer.^35–37^ To further explore the role of the adenine moiety in the substrate recognition, we evaluated how replacing adenine with alternative nitrogenous bases, such as cytosine (12) or guanine (13) affects the interaction with the enzyme. Finally, the 2′-modified PAP analogs 14–16 were prepared as potential ST inhibitors to probe how essential this position is for cofactor recognition.

**Fig. 3:**
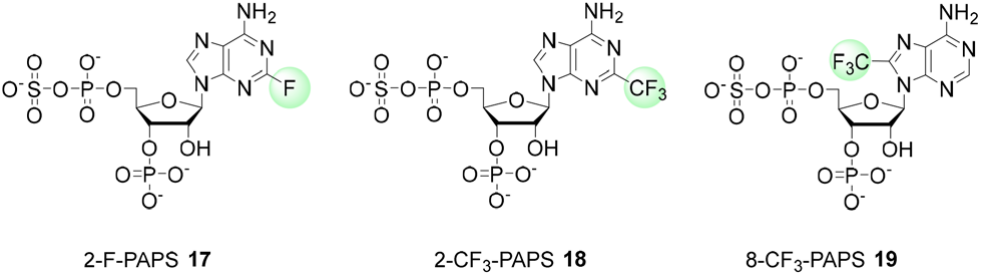
Structures of fluorinated PAPS analogs 17–19.

In addition to the synthesized set of compounds, fluorinated PAPS analogs 2-F-PAPS 17, 2-CF_3_-PAPS 18, and 8-CF_3_-PAPS 19 were used for monitoring ST activity by ^19^F NMR spectroscopy (Fig. 3).^38^

### Synthesis of analogs with modified phosphosulfate moiety 1–6

**Fig. 4:**
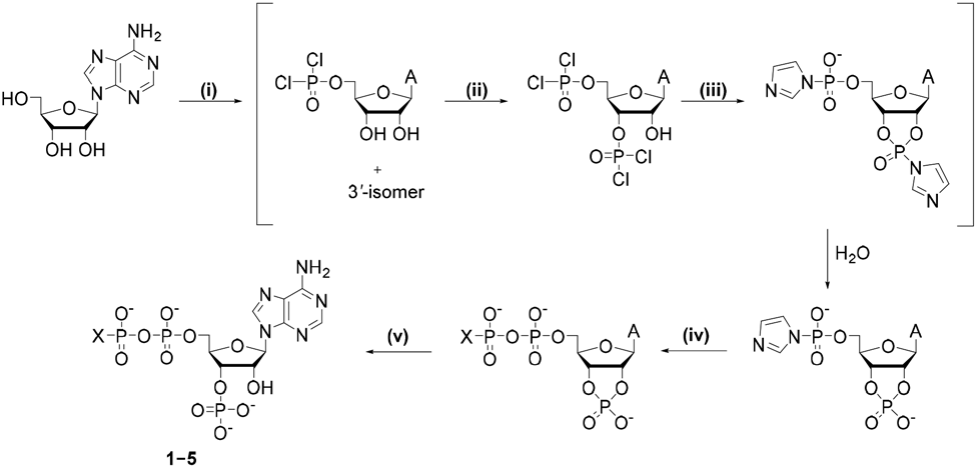
Synthesis of PAPP analogs 1–5. Conditions: (i) POCl_3_, PO(OCH_3_)_3_, 0 °C; (ii) POCl_3_, then precipitation with cold Et_2_O; (iii) Imidazole, PO(OCH_3_)_3_, then H_2_O; (iv) phosphate derivative, MgCl_2_ or ZnCl_2_, DMF; (v) RNAse T2 (50 mM ammonium acetate buffer, pH 7.3, 37 °C, 300 rpm), 24 h.

The synthetic pathway to compounds 1–5 was realized by a one-pot, three-step procedure, starting with a two-step phosphorylation of adenosine using POCl_3_, targeting the 5′- and 2′,3′-positions (see the SI for details).^39,40^ The progress of the reaction was monitored by reversed-phase high-performance liquid chromatography (RP-HPLC). The resulting bis(chlorophosphorylated) intermediate was hydrolyzed under alkaline conditions in the presence of excess imidazole and purified by ion exchange chromatography (DEAE Sephadex) to yield a PAP-imidazolide.^41,42^ The subsequent reaction with an organic salt of phosphate^41^ or an appropriate phosphate derivative^43,44^ in the presence of ZnCl_2_ in an aprotic solvent afforded cyclic intermediates in moderate yields (36–52%). The resulting products were purified by ion-exchange chromatography followed by semi-preparative RP-HPLC (Fig. 4 and Scheme S1). The final enzymatic hydrolysis by RNase T2 followed by RP HPLC purification led exclusively to the desired 3′-phospho isomers of PAPS analogs, compounds 1–5, in high yields (∼90%).^45^

Only bisphosphonate compound 6 was obtained by a different route. The phosphorylation of 2′,3′-cyclophosphoadenosine was performed using with CH_2_(POCl_2_)_2_, which was possible due to its higher reactivity compared to POCl_3_, providing the desired compound 6 in 6% yield (Scheme S2).^46^

### Synthesis of nucleobase-modified analogs 7–13

PAPP and PAPS analogs containing linkers of varying lengths at positions C8 and N6 of the adenine (7–11) and adenine replaced by another nucleobase (12,13) were synthesized following essentially the same synthetic pathway as previously reported for unmodified PAPS^47^(Schemes S3–S6). The synthesis of modified adenosine nucleosides was performed starting from C8- or N6-chloropurine ribosides by nucleophilic substitution with appropriate amino-functionalized linkers. The nucleosides were subjected to a sequence of phosphorylation, coupling, and enzymatic cleavage steps similar to those previously described for PAPP and PAPS analogs, yielding the target compounds in moderate to good yields 7–11 (37–72%).

During the synthesis of cytidine and guanosine PAP analogs (12 and 13), a higher tendency for decomposition of the dichlorophosphorylated intermediates was observed during reaction work-up in cold diethyl ether. Thus, we attempted to optimize the conditions using a base that would act as a nucleophile, inducing spontaneous cyclization of the phosphate in the 2′,3′ position (to avoid the isolation of a mixture of 2′/3′ isomers). The use of imidazole led to the expected P-imidazolide derivatives; however, the following hydrolysis of these intermediates occurred along with the hydrolysis of 2′,3′-cyclophosphate to an undesirable mixture of 2′/3′-phosphates. The issue was resolved by using a more reactive 1,2,4-triazole, which could easily be removed from the 5′-phosphate under mildly acidic conditions while keeping the 2′,3′-cyclophosphate intact. Following this route compounds 12 and 13 were synthesized in good yields (39% and 37%, respectively) and then subjected to the enzymatic cleavage reaction (Scheme S6).

### Synthesis of phosphoribose-modified PAP analogs 14–16

The preparation of 2′-F-PAP (14) included a two-step phosphorylation of commercially available 2′-fluoro-2′-deoxyadenosine, followed by hydrolysis and purification by RP HPLC with a yield of 10% (Scheme S7).

Phosphorothioate analogs of PAP, P_S_AP_S_ (15) and 2′-O-pentynyl-P_S_AP_S_ (16), were synthesized starting from adenosine and 2′-O-(pent-4-yn-1-yl)adenosine, respectively. Initially, O-alkylation with 5-chloro-1-pentyne occurred at the 2-position of adenosine (18% yield). Both nucleosides were reacted with diphenyl H-phosphonate under an inert atmosphere to introduce H-phosphonate groups at the 5′-O and 3′-O positions.^48^ Then, the products were oxidized with elemental sulfur in the presence of bis(trimethylsilyl)acetamide (BSA) (yields: 22% for 15; 85% for 16) (Schemes S8 and S9).^49^

### MST-based assay development

Next, we aimed to evaluate the binding properties of the synthesized PAP and PAPS analogs. As sufficiently high-throughput, cost-effective, and sample-saving methods for STs have not yet been developed to the best of our knowledge, we decided to develop a microscale thermophoresis-based (MST) binding assay for STs. The MST assays monitor ligand-dependent changes in the migration of fluorescently labeled molecules across a temperature gradient.^50–62^ As a model sulfotransferase for the assay development, we selected the plant sulfotransferase AtSOT18 due to its high stability and well-characterized properties. The protein was equipped with a fluorescent label by attaching it to the N-terminal HisTag.

Taking previously reported conditions^63,64^ as an initial reference point, optimal experimental conditions for our study were established through preliminary experiments performed in different binding buffers with varying pH and ionic strength using PAP as a model ligand.

**Fig. 5:**
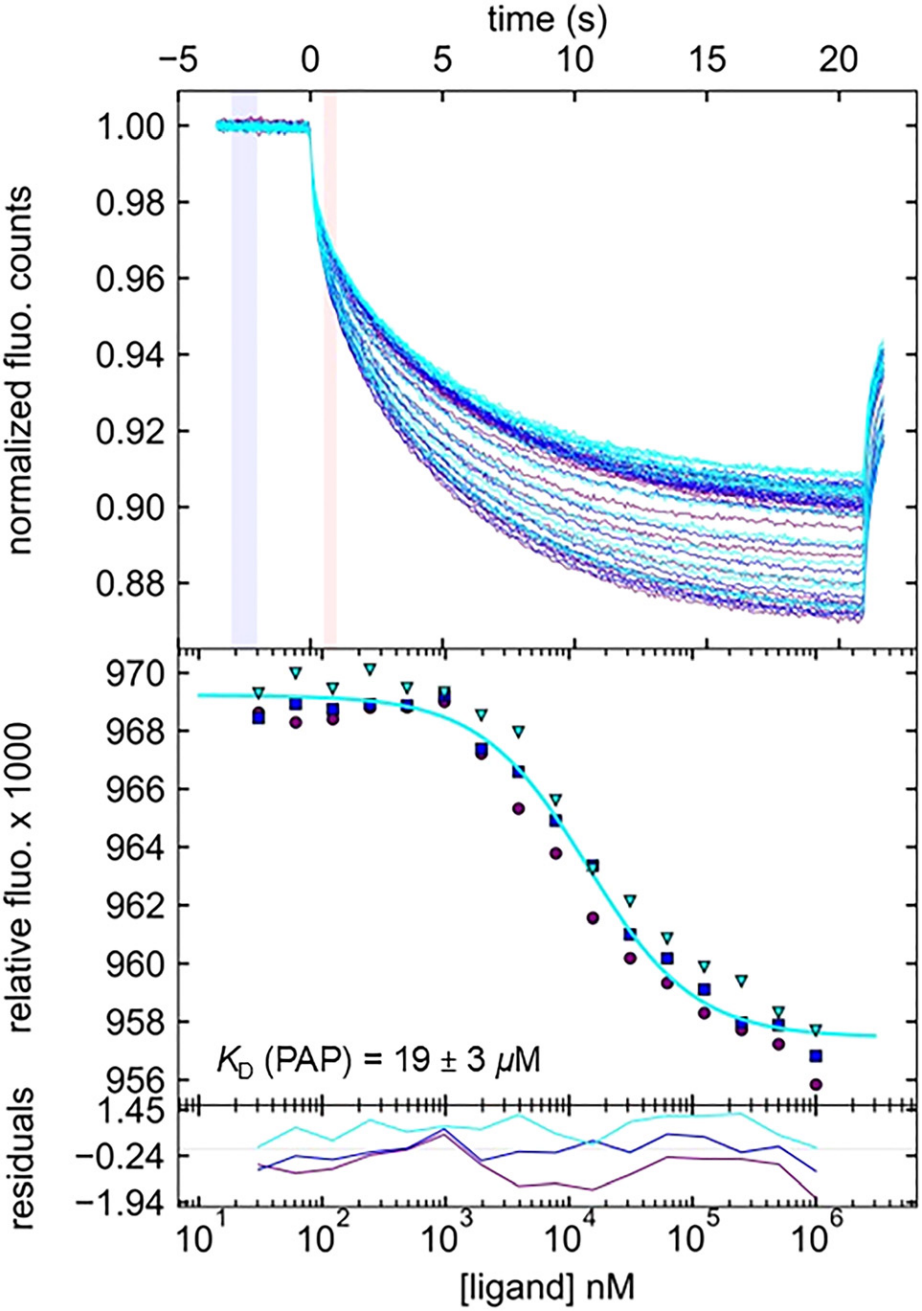
An MST binding curve of PAP for His_6_-ATSOT18 under optimal conditions (50 mM HEPES buffer, pH 6.5, 100 mM KCl, 0.05% Tween-20, 25 °C). The upper panel shows individual thermophoretic curves with “hot” and “cold” regions used for *K*_D_ determination marked in red and blue, respectively. The lower panel shows the binding curve determined from replicate experiments.

The highest stability of His_6_-ATSOT18 (determined by differential scanning fluorimetry DSF), combined with a well-defined binding curve (determined by MST), was found in 50 mM HEPES, pH 6.5, 100 mM KCl, and 0.05% Tween-20 buffer (Fig. 5). Under the optimized conditions, the dissociation constant, *K*_D_ determined for PAP was 19 ± 3 µM (Table 1, entry 4), which is comparable to the inhibition constant previously obtained for this enzyme by two-dimensional titration (*K*_I_ = 3.0 ± 1.7 µM; the difference can be attributed to various pH and buffering conditions).^65^ A similar measurement was also performed at a saturating concentration of the specific substrate, desulfosinigrin. Interestingly, the addition of desulfosinigrin resulted in more than 30-fold decrease in *K*_D_ (Table 1, entry 5). This observation suggests a functional coupling between substrate and cofactor binding. However, the structural basis of this effect remains to be determined. The dissociation constant for PAPS (13.0 ± 2.0 µM) was only investigated under non-substrate addition conditions (Table 1, entry 7; literature data: 4.2 ± 2.6 µM^65^), since measurements with the addition of substrate would result in an enzymatic reaction, making such experiments not possible. Comparable values for the PAP and PAPS binding data correspond well with the existing data from previous enzymatic and binding studies, which generally show little difference in affinity between PAPS and PAP. It was also found that ligand binding additionally stabilized the protein (Fig. S5).

**Table 1:** Dissociation constant *K*_D_ values for His_6_-ATSOT18^a^ and His_6_-SULT1A^b^ with various ligands determined by MST-based assay

Entry	Ligand	His_6_-ATSOT18	*n*	His_6_-SULT1A3	*n*
		*K*_D_ [µM]		*K*_D_ [µM]	
1	AMP	1900 ± 1300	2	200 ± 100	2
2	ADP	1000 ± 500	3	13 ± 7	2
3	ATP	68 ± 13	3	n.d.	—
4	PAP	19 ± 3	3	0.7 ± 0.2	6
5^c^	PAP	0.5 ± 0.12	4	n.d.	—
6	cPAP	130 ± 80	2	n.d.	—
7	PAPS	13 ± 2	3	30 ± 16	3
8	1	12 ± 2	3	3 ± 2	2
9^c^	1	2.4 ± 0.9	3	n.d.	—
10	2	7.8 ± 1.2	3	28 ± 12	2
11	3	46 ± 2	2	14 ± 11	3
12^c^	3	23 ± 8	3	n.d.	—
13	4	90 ± 40	2	70 ± 20	2
14	5	180 ± 60	2	n.d.	—
15	6	280 ± 140	2	40 ± 30	3
16	7	16 ± 2	3	n.d.	—
17	8	20 ± 6	2	70 ± 40	3
18	9	8.4 ± 1.6	2	n.d.	—
19	10	22 ± 9	4	n.d.	—
20	11	93 ± 10	4	390 ± 150	3
21	12	2200 ± 500	3	n.d.	—
22	13	670 ± 150	3	n.d.	—
23	14	17 ± 3	3	1000 ± 1.1	2
24	15	21 ± 8	2	n.d.	—
25	16	38 ± 10	3	20 ± 5	3
26^c^	16	29 ± 13	2	n.d.	—
27	17	43 ± 10	3	n.d.	—
28	18	390 ± 90	3	n.d.	—
29	19	130 ± 31	3	n.d.	—

a Conditions for His_6_-ATSOT18 : 50 mM HEPES, pH 6.5, 100 mM KCl, 0.05%.

b Conditions for His_6_-SULT1A3: PBS-T buffer.

c Experiment was carried out with the addition of desulfosinigrin.

Next, the human sulfotransferase labeled with a fluorescent HisTag, SULT1A3, was examined to determine whether the described MST method is applicable to study other ST enzymes. Although SULT1A3 is known as a thermolabile human phenol sulfotransferase, its biological role remains highly important. Therefore, we sought to compare results across enzymes to assess the specificity and universality of the effects of the modifications on interactions with ST. Based on results from the DSF method, PBS-T buffer was selected as the optimal, yielding the highest protein stability (Fig. S4). Under the optimized conditions, the *K*_D_ for PAP was 0.7 ± 0.2 µM (Table 1, entry 4), whereas from literature data for SULT1A2, a human cytosolic sulfotransferase, the *K*_D_ was 0.3 µM.^65^ As expected, the His_6_-SULT1A3 enzyme was found to be much more sensitive to small changes in experimental conditions, leading to multiple aggregates often forming during measurements and prolonged storage. Poor protein stability was further exacerbated by certain ligands, rendering some measurements impossible and highlighting protein stability as the key limitation of the developed MST-based assay. These observations are consistent with the thermolabile nature of SULT1A3 and support the use of more stable ST enzymes in future studies.

### Evaluation of the compound library by an MST-based assay

Since the results obtained were in relatively good agreement with previously reported values, the developed method was employed to evaluate the contributions of individual elements of the PAPS structure to affinity for STs. To that end, we first determined the *K*_D_ values for all synthesized compounds 1–19 for His_6_-ATSOT18, along with several natural compounds that served as references (Table 1). These measurements provided valuable insights into the structure–activity relationship and the role of the 3′-phosphate group in stabilizing the enzyme complex.

The results for His_6_-ATSOT18 showed that PAP and PAPS are among the strongest binders from all tested compounds (Table 1). One of the comparably strong binders was an ADP derivative bearing an additional phosphate group at the 3′-position, PAPP 1 (Table 1, entry 8). Interestingly, this finding suggests that the enzyme does not differentiate between 5′-phosphosulfate and 5′-diphosphate moieties, making PAPP and its derivatives close structural but non-functional mimics of PAPS. Furthermore, a comparative analysis of binding data for PAPS, PAPP, PAP, AMP, ADP, and ATP generalizes the finding that the 5′-terminal sulfate/phosphate group at the β-position makes a modest contribution to the stabilization of the complex (compare *e.g.*Table 1, entries 7 and 8), whereas the presence of 3′-phosphate increases the binding affinity by 2 orders of magnitude (compare *e.g.*Table 1, entries 2 and 8). These findings suggest one possible explanation for the conservation of the 3′-phosphate moiety in PAPS,^66^ namely its contribution to binding affinity and molecular recognition by sulfotransferases. The fact that the ST is unable to “distinguish” between 5′-phosphosulfate and 5′-diphosphate residues at the molecular level suggests that the additional 3′-phosphate is a major structural determinant of high-affinity binding of the cofactor to sulfotransferases. We hypothesize that the 3′-phosphate enables differentiation between the endogenous phosphosulfate cofactor and ADP/ATP, especially since the latter are typically present in the cells at high (millimolar) concentrations. Similar results from measurements performed for His_6_-SULT1A3 on a smaller subset of compounds are consistent with this hypothesis (Table 1).

The difference in affinity between ADP and PAPP allowed us to estimate the stabilization energy contributed to the complex by 3′-phosphate, which was 2.60 kcal mol^−1^, corresponding approximately to the energetic contribution typically associated with a hydrogen bond.^67^ Interestingly, the presence of desulfosinigrin, significantly decreased *K*_D_ value (Table 1, entry 9). The addition of another phosphate group, PAPPP 2, resulted in slightly lower dissociation constant than for the natural cofactor (Table 1, entry 10), indicating an increase in the affinity for the protein binding pocket by an additional 5′-phosphate. Surprisingly, replacing sulfate with a fluorophosphate group in 3, which is an isosteric and isoelectronic analog of sulfate, showed a 4-fold decrease in affinity compared to PAPS (Table 1, entry 11), and an analogs two-fold decrease in the presence of desulfosinigrin (Table 1, entry 12). Analysis of compounds containing alkyne groups, such as C-ethynyl 4 or propargyl 5, showed very low affinity (Table 1, entries 13 and 14). The presence of a methylene bisphosphonate residue, which is an analog of diphosphate with a CH_2_ group in place of the bridging oxygen atom, resulted in a greater than 20-fold reduction in binding affinity for 6 to His_6_-ATSOT18 (Table 1, entry 15), rendering this modification one of the few that seem to strongly disrupt the binding. Overall, these studies show that small modifications, isosteric to sulfate or slightly larger, do not significantly affect binding affinity, either in the presence or absence of the co-substrate, whereas larger modifications, such as phosphate with clickable moieties, disrupt binding to a greater extent.

The next set of tested compounds was nucleobase-modified analogs 7–11 (Table 1, entries 16–20). Introduction of azidoethyl residue at the C8 position (compounds 7, 8) resulted in affinities similar to those observed for PAP and PAPS (Table 1, entries 16 and 17). In contrast, introduction of the same azidoethyl substituent at the N6 position in 9 resulted in a markedly higher affinity (Table 1, entry 18). However, a longer linker, 8-azido-3,6-dioxoctane-1-amine, in 10 showed a 2-fold decrease in affinity (Table 1, entry 19), whereas its sulfate analog 11 showed four times lower affinity (Table 1, entry 20). As we expected, the effect of nucleobase modifications on affinity depends both on the size of the substituent/linker, and on its position, wherein N6 positions seemed to be slightly more preferred over C8. Additional important information in this section was provided by analysis of fluorinated PAPS analogs, such as 2-F-PAPS 17, 2-CF_3_-PAPS 18, and 8-CF_3_-PAPS 19 (Fig. 5, Table 1, entries 27–29). A small modification at the C2 position, such as fluorine, did not significantly affect the affinity of His_6_-ATSOT18 (Table 1, entry 27). Whereas the larger CF_3_-group in 18,19 caused a 10–30-fold decrease compared to unsubstituted PAPS (Table 1, entries 28 and 29). Replacing adenine with cytosine (12) or guanine (13) resulted in a significantly weaker affinity for His_6_-ATSOT18 compared to PAP, indicating a very strong preference for the adenine base in the cofactor structure (Table 1, entries 21 and 22).

In the series of compounds modified at the 2′ position 14–16, only 2′-F-PAP 14 exhibited an affinity comparable to that of PAP, indicating that the fluorine substitution does not interfere with binding (Table 1, entry 26). In contrast, both sulfur-containing analogs 15, 16 displayed slightly reduced affinity for His_6_-ATSOT18, regardless of the presence of desulfosinigrin (Table 1, entries 27–29).

The results for another tested enzyme, human His_6_-SULT1A3, are also presented in Table 1. Owing to the reduced stability of this enzyme, not all ligands were investigated. Any structural modifications to PAP resulted in at least a 10-fold decrease in binding affinity. The absence of a phosphate group at the 3′ position significantly reduced protein stability, as indicated by thermophoretic curves showing aggregate formation. Only replacing the sulfate group with a phosphate 1 or fluorophosphate group 3 resulted in stronger binding for His_6_-SULT1A3 than PAPS but not than a natural inhibitor PAP (Table 1, entries 8, 11). Other modifications within the phosphosulfate or nucleobase moiety showed comparable affinity to PAPS (2, 6, 16, Table 1, entries 10, 15, 25) or a significant decrease (4, 8, 9, 11, entries 13, 17, 18, 20).

The introduction of fluorine into the 2′ position in 2′-F-PAP 14 resulted in a more than 1000-fold decrease in ligand affinity compared to PAP (Table 1, entry 23). This observation suggests that interactions involving the native 2′-hydroxyl group may contribute to ligand recognition by SULT1A3, although the structural basis remains unclear. Surprisingly, 2′-*O*-pentynyl-P_S_AP_S_16 showed a similar *K*_D_ value to PAPS (Table 1, entry 25).

Summarizing the results obtained for two sulfotransferases labeled with a fluorescent HisTag, plant AtSOT18 and human SULT1A3, the available data suggest that modifications disrupting the stabilizing interactions involving the 3′-phosphate group, as in cPAP, AMP, ADP and ATP, lead to a decrease in ligand affinity for both enzymes. In contrast, the exchange of sulfate for phosphate appears to be an acceptable modification in general. Compounds modified at the N6, C2, and C8 positions of adenine are usually considered binders, although the specific effects depend strongly on substituent size and position. However, we found these positions dispensable, except for the azidoethyl N6 modification in 9, which exhibits preferential binding to AtSOT18. The decisive preference for the adenine base itself is also evident. Functionalization of 2′-position of the ribose or introduction of phosphorothioate moieties did not significantly affect binding to His_6_-ATSOT18 but is crucial for His_6_-SULT1A3, showing that these sites enable modifications directed at tailoring specificity towards particular STs.

### Activity-based screening using ^19^F NMR

Since the MST-binding assay was limited by protein stability, we turned to an orthogonal, functional assay. To that end, we chose a recently developed method for monitoring ST activity by ^19^F NMR spectroscopy using fluorinated PAPS analogs such as 2-F-PAPS 17, 2-CF_3_-PAPS 18, and 8-CF_3_-PAPS 19 (Fig. 3).^38^ Substrate properties of these compounds were previously investigated with plant AtSOT18 and human SULT1A3, and all three compounds were found to be promising molecular tools for studying STs, *e.g.* activity-based inhibitor screening against therapeutically relevant sulfotransferases, or assaying the ST activities in complex biological mixtures. Although 2-F-PAPS 17 serves as a substrate for both enzymes, it contains only one fluorine atom as the ^19^F label, and spectra obtained using 2-F-PAPS 17 exhibit a lower signal-to-noise ratio compared to those obtained with 2-CF_3_-PAPS 18 or 8-CF_3_-PAPS 19 at the same concentration. Therefore, we decided to focus on the assay using CF_3_-substituted analogs as a valuable tool for screening modified PAPS analogs 1–16 as potential inhibitors of sulfuryl group transfer.

**Fig. 6:**
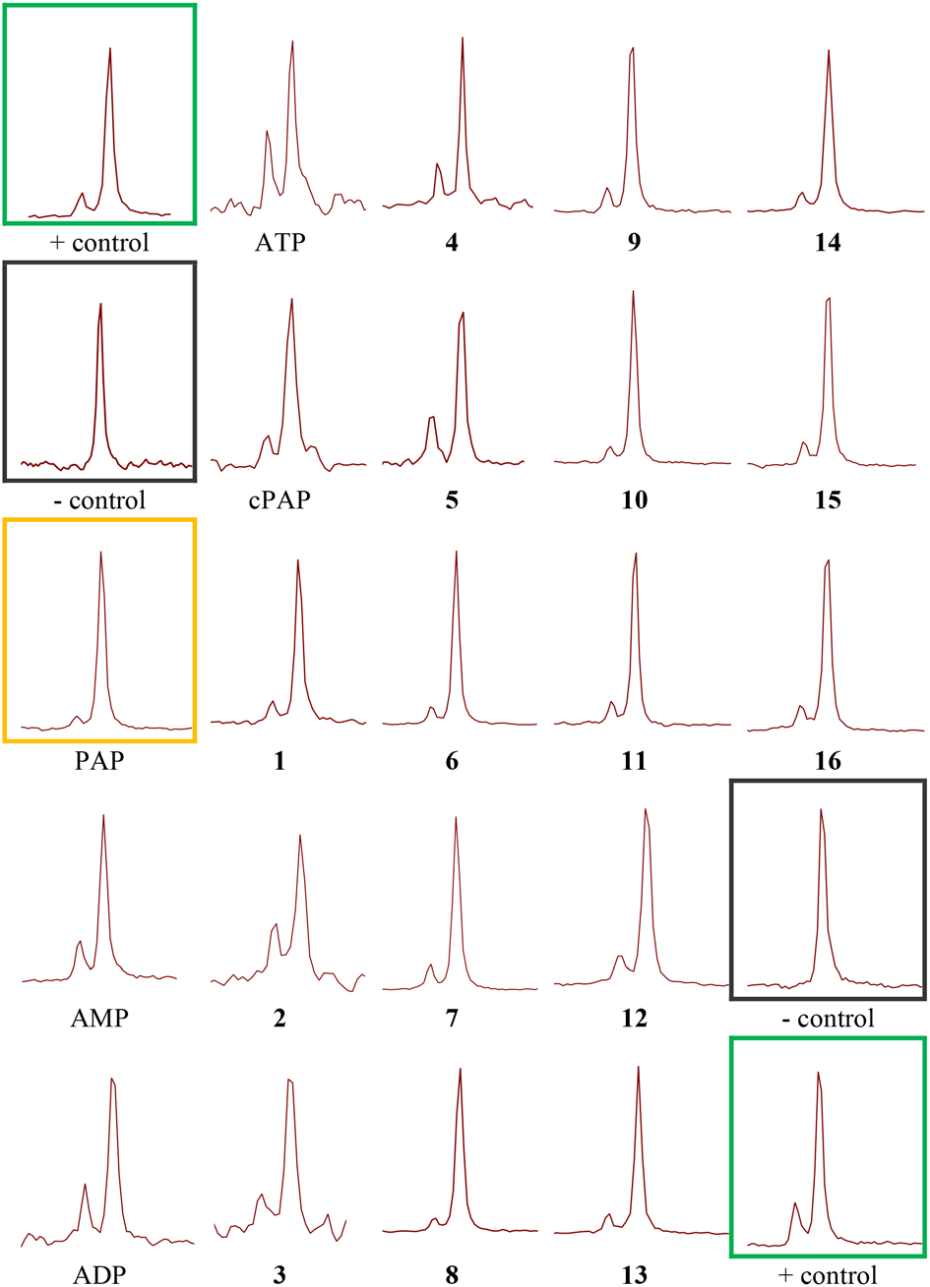
^19^F NMR-based screening of PAPS analogs against AtSOT18. The *y*-axis reports ^19^F NMR Signal Intensity. Samples in green frames: reactions without an inhibitor (positive controls). Samples in black frames: reactions without an enzyme (negative controls). Sample in yellow frame: reaction in the presence of PAP (inhibition control).

First, we screened PAPS analogs as potential AtSOT18 inhibitors in the presence of 2-CF_3_-PAPS 18. Distinct ^19^F signal-separation between 2-CF_3_-PAPS (right peak) and the reaction-formed 2-CF_3_-PAP (left peak) was observed for all reactions under previously optimized conditions (83 mM Tris, pH 9.0, 9.2 mM MgCl_2_ with 10% D_2_O) at the 40 min time point at 37 °C (Fig. 6; conversions 6–28%). Control reactions included samples without an inhibitor, without a protein, and with the addition of a natural ST inhibitor (PAP). Most of the tested ligands 1–16 afforded conversions in the range of 12–18%, whereas the natural ST inhibitor PAP resulted in 9% conversion (Table 2, entry 2). Among the tested analogs, compound 8 emerged as the most promising hit in the screening assay, limiting PAPS-to-PAP conversion to 6% under the assay conditions (Table 2, entry 14).

**Table 2:** Inhibitory activity assay against AtSOT18^a^ and SULT1A3^b^

Entry	Ligand	AtSOT18 conv., %	SULT1A3 conv., %
1	—	21	48
2	PAP	9	28
2	AMP	21	44
3	ADP	21	41
4	ATP	28	25
6	cPAP	14	20
7	1	13	14
8	2	13	21
9	3	17	40
10	4	16	17
11	5	27	35
12	6	12	36
13	7	13	3
14	8	6	18
15	9	15	36
16	10	12	28
17	11	13	25
18	12	18	32
19	13	13	36
20	14	12	20
21	15	12	27
22	16	15	17

a Reaction conditions for AtSOT18 – 200 µM 2-CF_3_PAPS 18, 200 µM dopsinigrin, 35 µM inhibitor (0.96 µL of the 10 mM stock solution), 100 nM enzyme in 83 mM Tris, pH 9.0, 9.2 mM MgCl_2_ with 10% D_2_O at 37 °C.

b Reaction conditions for SULT1A3 – 200 µM 8-CF_3_-PAPS 19, 200 µM dopamine, 35 µM inhibitor (0.96 µL of the 10 mM stock solution), 100 nM enzyme in 6.7 mM K_2_HPO_4_, pH 7.4, with 10% D_2_O at 37 °C.

Interestingly, compound 8 exhibited only moderate affinity in the MST assay. This observation highlights that equilibrium binding affinity does not necessarily correlate directly with the apparent inhibitory effects observed in the ^19^F NMR assay. Given the exploratory nature of the screening experiment and the complexity of the underlying enzymatic system, the mechanistic basis of this discrepancy remains unclear and warrants further investigation.

**Fig. 7:**
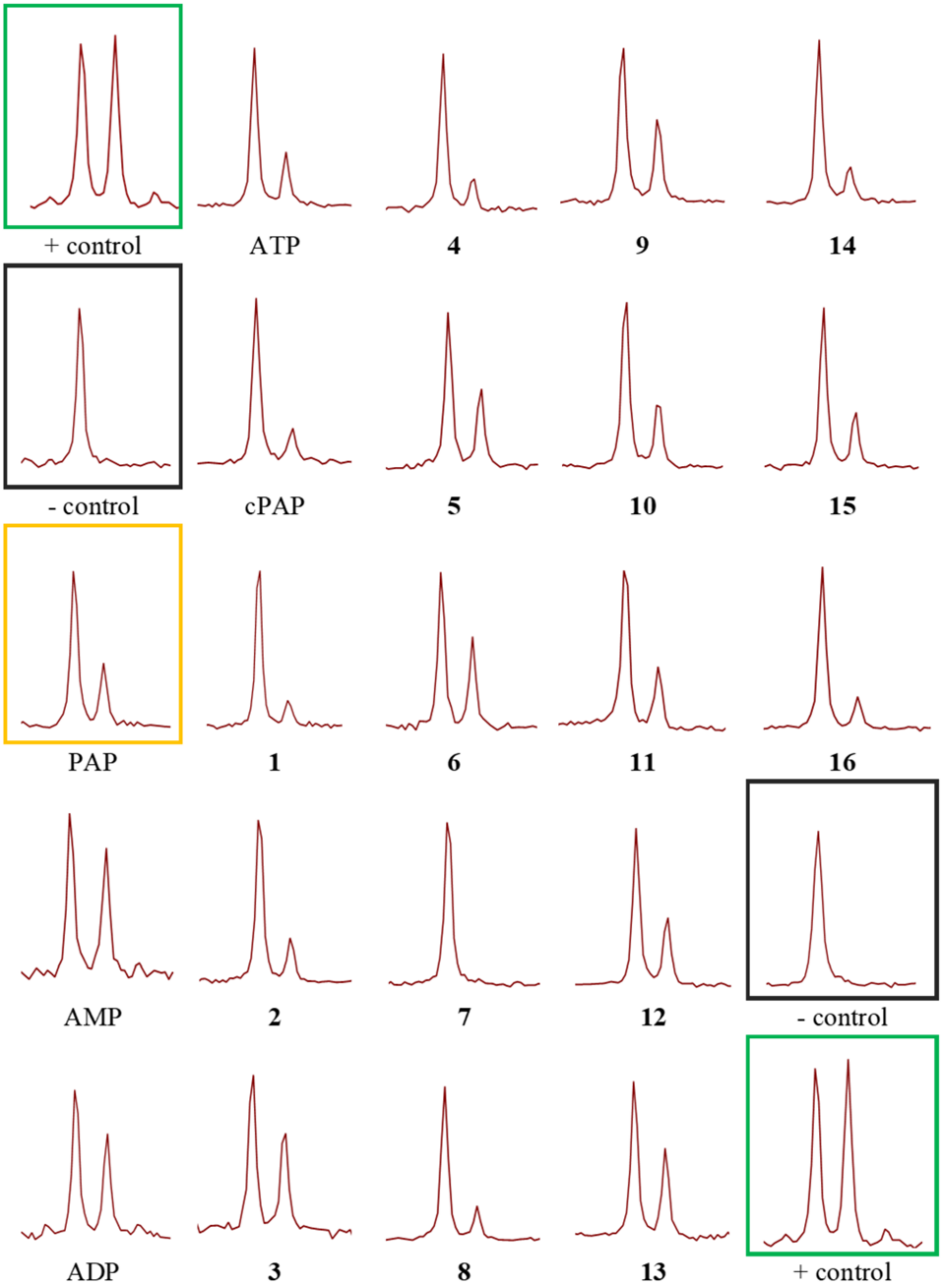
^19^F NMR-based screening of PAPS analogs against SULT1A3. The *y*-axis reports ^19^F NMR Signal Intensity. Samples in green frames: reactions without an inhibitor (positive controls). Samples in black frames: reactions without an enzyme (negative controls). Sample in yellow frame: reaction in the presence of PAP (inhibition control).

SULT1A3-catalyzed reactions were then performed for all ligands 1–16 in the presence of 8-CF_3_-PAPS 19 in 6.7 mM K_2_HPO_4_, pH 7.4, with the addition of 10% D_2_O at 37 °C. ^19^F NMR spectra recorded after 40 min represent two signals, where the left signal corresponds to 8-CF_3_-PAPS and the right signal to 8-CF_3_-PAP (Fig. 7, conversions 3–48%). Identical control reactions were conducted, and in the absence of an inhibitor, the reaction proceeded to 48% conversion. This allowed for a clear differentiation of the inhibitory effects of ligands 1–16 (Table 2, last column). As a result, compound 7, a phosphate analog of 8, exhibited the most potent inhibitory activity in the current study with only 3% conversion PAPS to PAP (Table 2, entry 13).

Overall, our results demonstrate that the ^19^F NMR assay provides a useful orthogonal screening method for identifying compounds that may modulate sulfotransferase activity and therefore merit more detailed kinetic characterization.

## Conclusions

A library of 16 chemically modified PAPS and PAP analogs was created to evaluate their ability to bind and inhibit model STs: plant AtSOT18 and human SULT1A3. To better understand the structure–activity relationship in PAPS–ST interactions, different positions of the PAPS/PAP moieties were modified. These included replacement of the 5′-phosphosulfate group with alternative moieties, such as diphosphate, fluorophosphate, bisphosphonate, and others; modifications of the nucleobase, either through functionalization of the adenosine with clickable linkers or by replacing the adenine with cytosine or guanine; and functionalization at the 2′-position of the ribose or introduction of phosphorothioate groups. Structurally similar natural nucleotides were also investigated.

Microscale thermophoresis was used to evaluate the binding properties of novel compounds, revealing that the 3′-phosphate group appears to play a major role in enhancing binding affinity for STs, whereas the 5′-phosphosulfate and diphosphate moieties contribute only minimally. This suggests that the 3′-phosphate in PAPS may serve as an important structural determinant likely contributing to affinity-based differentiation between adenosine 5′-phosphosulfate and 5′-diphosphate compounds. Additional 5′-phosphorylation slightly enhanced affinity for both enzymes. Therefore, PAPP, PAPPF and other non-functional phospho-analogs of PAPS, constitute particularly suitable ligands for crystallographic studies and other structural studies due to their increased stability compared to PAPS.

Nucleobase and ribose substitutions were also, to a large extent, tolerated by the enzymes, but the particular effects, not surprisingly, strongly depended on the position and modification type. In a series of compounds synthesized as potential ST inhibitors with modifications at the 2′ position, no substantial effect on binding or activity was observed.

Finally, a strong preference for the adenine base in the cofactor structure was also revealed through base replacement.

Interestingly, the ^19^F NMR-based identified C8-(1-amino-2-azidoethyl)-substituted compounds (7,8) as strong inhibitors of both AtSOT18 and SULT1A3, highlighting their potential for further development as molecular tools targeting ST-enzymes. Taken together, these results establish a foundation for developing PAPS-derived molecular tools and provide a starting point for future structural and enzymological investigations into sulfotransferase recognition. Further research in biologically relevant experimental setups is needed to fully assess the potential of the identified compounds.

## Author contributions

M. M., A. M.-C., and J. K. designed the study, A. M.-C. and M. M. performed the syntheses, A. M.-C. and M. M. performed experiments, M. Ch. and T. S. provided resources, J. W. M. contributed new concepts, J. K., and J. J. supervised experiments, J. K. provided funding, M. M. and J. K. wrote the first draft of the article. All authors wrote, edited, and approved the final version of the article.

## Conflicts of interest

There are no conflicts to declare.

## Supplementary Material

CB-OLF-D6CB00094K-s001

## Data Availability

The data supporting the findings of this study are available within the main article and its supplementary information (SI). Supplementary information is available DOI: https://doi.org/10.1039/d6cb00094k. Raw data are available from the corresponding author upon reasonable request.
