# Supplementary material for: Probing sulfotransferase binding and inhibition with synthetic PAPS analogs reveals the role of the 3′-phosphate and informs molecular tool design
Source: RSC Chem Biol. 2026 Jun 19. Online ahead of print. doi: 10.1039/d6cb00094k (PMC13321160; doi:10.1039/d6cb00094k)
Supplement: CB-OLF-D6CB00094K-s001 [file CB-OLF-D6CB00094K-s001.pdf]

Supplementary Information (SI) for RSC Chemical Biology.
This journal is © The Royal Society of Chemistry 2026

Supporting Information

Probing Sulfotransferase Binding and Inhibition with Synthetic PAPS Analogs Reveals the Role of the 3′-Phosphate and Informs Molecular Tool Design

Maria Matveeva, [a,#] Agnieszka Młynarska-Cieślak, [a,#] Mikołaj Chromiński, [b] Tomasz Spiewla, [b] Jonathan W. Mueller, [c] Jacek Jemielity [b] and Joanna Kowalska*[a]

[a] *Division of Biophysics, Institute of Experimental Physics, Faculty of Physics, University of Warsaw, Pasteura 5, 02-093 Warsaw, Poland*

[b] *Centre of New Technologies University of Warsaw, Banacha 2c, 02-097 Warsaw, Poland*

[c] *Department of Metabolism and Systems Science, School of Medical Sciences, College of Medicine and Health, University of Birmingham, Birmingham, UK*

[#]*These authors contributed equally to the work*

**E-mail: jkowalska@fuw.edu.pl*

1. General information 3

2. AtSOT18 and SULT1A3 expression and purification 5

3. Synthesis of 3'-phosphoadenosine 5'-phosphate 7

4. General Procedure for the Synthesis of PAPP analogues **1**–**6** 8

   a. 3′-Phosphoadenosine 5′-diphosphate **1** 9
   b. 3′-Phosphoadenosine 5′-triphosphate **2** 10
   c. 3′-Phosphoadenosine 5′-fluoro diphosphate **3** 11
   d. 3′-Phosphoadenosine 5′-phosphate-C-ethynyl phosphonate **4** 12
   e. 3′-Phosphoadenosine 5′-propargyl diphosphate **5** 13

5. Synthesis of 3′-phosphoadenosine 5′-methylene bis-phosphonate **6** 14

6. General Procedure for the Synthesis of C8-modified analogues **7**,**8** 15

   a. 3′-Phospho-8-(1-amino-2-azidoethyl) adenosine 5′-phosphate **7** 16
   b. 3′-Phospho-8-(1-amino-2-azidoethyl) adenosine 5′-phosphosulfate **8** 17

7. Synthesis of 3′-phospho-6-(1-amino-2-azidoethyl) adenosine 5′-diphosphate **9** 18

8. General Procedure for the Synthesis of N6-modified analogues **10**,**11** 20

   a. 3′-Phospho-6-(8-azido-3,6-dioxyoctano-1-amino) adenosine 5′-diphosphate **10** 20
   b. 3′-Phospho-N6-(1-amino-8-azido-3,6-dioxyoctano) adenosine 5′-phosphosulfate **11** 22

9. General Procedure for the Synthesis of **12**,**13** 23

   a. 3’-Phosphocytidine 5’-diphosphate **12** 23
   b. 5’-Diphosphate -3’-phosphoguanosine **13** 25

10. Synthesis of 5’,3’-diphosphate 2’-fluoro adenosine **14** 25

11. Synthesis of 5’,3’-adenosine dithiophosphonate **15** 26

12. Synthesis of 5’,3’-dithiophosphate-2-O-pentynyl adenosine **16** 27

13. General Procedure for the Synthesis of Fluorinated PAPS **17**–**19** 29

   a. 3’-Phospho-2-(fluoro) adenosine 5’-phosphosulfate **17** 30
   b. 3’-Phospho-2-(trifluoromethyl) adenosine 5’-phosphosulfate **18** 31
   c. 3’-Phospho-8-(trifluoromethyl) adenosine 5’-phosphosulfate **19** 32

14. HPLC assay 32

15. Microscale thermophoresis assay 33

   a. MST-based assay development 33
   b. Evaluation of the compound library by an MST-based assay 37

16. ^{19}F NMR-Monitored Sulfotransferase Experiments Assay 39

   a. Screening with 2-CF_3-PAPS for AtSOT18 39
   b. Screening with 8-CF_3-PAPS for SULT1A3 39

References 40

1. General information

The synthesis of fluorinated PAPS used as a control for reactions with sulfotransferases was carried out according to known procedure described by Kowalska et al.[1, 2] Imidazole fluorophosphate for derivative **3** and ethynyl C-phosphonate for the synthesis of **6** were synthesised by P. Wanat.[3] Propargyl phosphate and 2′-O-(1-pent-5-yn) adenosine were obtained following the protocols described in Walczak et al.;[4] Jawalekar et al.[5] All other starting materials, solvents and chemical reagents were purchased from Merck, and POCH and used without further purification unless stated otherwise. Nucleosides (A, C, U, G and 2'-F-A) and desulfosinigrin were purchased from Sigma-Aldrich. Dopamine hydrochloride was purchased from Alfa Aesar. RNase T2 was purchased from Mobitech (Germany). All cyclic parent compounds have the same numbering including the prime symbol **1′–19′**. The structures of all synthesized compounds **1**–**19**, with the exception of compound **11**, were confirmed by NMR spectroscopy and high-resolution mass spectrometry (HR-MS). Compound **11** could not be fully characterized due to the insufficient amount of material obtained.

For purification and analytic characterisations, the following devices were used:

Ion-exchange chromatography was performed by ion-exchange chromatography on a DEAE Sephadex A-25 (HCO_3^- form) columns. A column was loaded with the reaction mixture and washed thoroughly with water until the eluate did not precipitate with 1% $AgNO_3$ solution (to remove residual solvents and non-binding reagents). Nucleotides were eluted using a 0 to 0.8 M and 0 to 1 M linear gradient of triethylammonium bicarbonate (TEAB) in deionized water for imidazole derivatives and final compounds respectively. Collected fractions were analyzed spectrophotometrically at λ = 260 nm and by RP-HPLC. Fractions containing the desired product were combined and concentrated under reduced pressure with repeated additions of ethanol (96%) and acetonitrile, to decompose TEAB and to remove residual water, respectively. The product was precipitated with acetonitrile. The resulting compounds were isolated as triethylammonium salts. Yields were calculated on the basis of optical density units (OD = volume in mL × absorbance of the solution) of the isolated products and corresponding starting materials (nucleotides or nucleotide P-imidazolide derivatives), except when described otherwise. The extinction coefficient for 2-F-adenosine **17** was the same as for non-modified adenine, 15020 (ε, mL/mmol/cm). For the parent CF_3-substituted nucleosides, as for

2-CF_3-adenosine **18** and 8-CF_3-adenosine **19,** the extinction coefficients were previously determined as 10660 and 11392 (ε, mL/mmol/cm), respectively.[2]

The semi-preparative RP HPLC purification was performed using a Clarity 5 μm Oligo-RP LC column (150×10 mm, flow rate 5.0 mL min–1) with UV detection at 254 nm and isocratic gradient (for **1-17**) of buffer A (0.05 M triethylammonium acetate buffer, pH 7) or linear gradient from buffer A to 50% of buffer B (1:1 of ACN : 0.1 M buffer A) over 60 min (for **18** and **19**). After repeated freeze-drying of the collected fractions, products were isolated as triethylammonium salts.

Analytical RP HPLC was performed using Gemini 3 μm NX-C18 LC column 110 Å (150×4.6 mm, flow rate 1 mL min^{-1}) for chemical reaction progress monitoring and enzymatic assays. A linear gradient from buffer A (ammonium acetate buffer, pH 5.9, 0.05 M) to 50% of buffer B (50:50 of methanol:buffer A) over 15 min was applied for compounds **1-17** or to 100% of buffer B for **18** and **19**.

^{1}H NMR spectra were recorded at room temperature on BRUKER AVANCE III HD spectrometer at 500 MHz (^{1}H NMR), 202 MHz (^{31}P NMR) and 471 MHz (^{19}F NMR). The data were interpreted in first order spectra. Chemical shifts δ are reported in parts per million (ppm) and for ^{1}H NMR were calibrated to sodium 3-trimethylsilyl-[2,2,3,3-D4]propionate (TSP) in D_2O, for ^{31}P NMR to H_3PO_4 (20%) in D_2O and for ^{19}F NMR to 0.1 M NaF in D_2O (δF = −121.5 ppm) as an external standard. The following abbreviations are used to indicate the signal multiplicity: s (singlet), d doublet), t (triplet), q (quartet), quin (quintet), sext (sextet), dd (doublet of doublet), dt (doublet of triplet), ddd (doublet of doublet of doublet), etc., br. s (broad signal), m (multiplet). Coupling constants (*J*) are given in Hz and refer to H, H-couplings. Signal assignments and identification were based on COSY spectra analysis. The NMR spectra were analyzed by MestReNova 12.0.1.

High resolution mass spectra (HR-MS) Mass spectra were recorded with LTQ Orbitrap Velos (Thermo Scientific) spectrometer.

Microscale thermophoresis (MST) measurements were performed using a Monolith NT (Nanotemper). Thermophoresis curves were analyzed using PALMIST92 software.[6]

2. AtSOT18 and SULT1A3 expression and purification

The sequence encoding SOT18 from Arabidopsis thaliana (Gene ID- 834749) in plasmid vector (pQE-30_His_AtSOT18) was obtained courtesy of Jutta Pappenbrock and Felix Hirschmann.[6] AtSOT18 protein (~42 kDa) flanked with His-tag on its N1-end was overexpressed in BL21 (DE3) RIL E.coli (Invitrogen) Prokaryotic system. 6xHis-AtSOT18 sulfotransferase expression was induced by 0.4 mM IPTG (Isopropyl-*β*-D-thiogalactoside) at optical density of 0.7 (bacterial culture) and the cells were further cultured for 16 h at 18°C. The cells were harvested and lysed in a buffer containing: 50 mM Na_2HPO_4 (pH 7.5), 300 mM NaCl, 30 mM imidazole, 0.1 mg/ml lysozyme and mixture of protease inhibitors (Aprotinin, Leupeptiun, Pepstatin, PMSF). The lysate was sonicated (15 min, Amplitude 50%, 15 s on/off) and centrifuged. The supernatant was then loaded on 2 x 5 ml HisTrap FFTM column (Cytiva) previously equilibrated with a buffer containing 50 mM Na_2HPO_4 (pH= 7.5), 300 mM NaCl, 30 mM imidazole. Protein AtSOT18 was eluted with a buffer containing 50 mM Na_2HPO_4 (pH= 7.5), 300 mM NaCl, 500 mM imidazole. The collected fractions were further purified on a Superdex 75 pg HiLoad 26/600 gel filtration column (Cytiva). Samples with 6xHis-AtSOT18 protein were concentrated to 30 μM, flash frozen and stored at - 80 °C in a buffer containing 20 mM Tris-HCl (pH 8.0), 100 mM NaCl, 1 mM DTT, 10% glycerol.

The human sulfotransferase SULT1A3 (Gene ID-6818) sequence in plasmid vector (pUC57-SULT1A3) was purchased from GenScript and cloned into the pET28 vector. The 6xHis-SULT1A3 cDNA was amplified by PCR using the sense primer 5'-TTAGGATCCATGGAACTGATTCAAGACACCAG-3' which inserts a BamHI site and the antisense primer 5'-GAGAAGCTTTTACAGTTCGCTACGAAAGCTCAG-3' which inserts a HindIII restriction site and plasmid pUC57-SULT1A3 as a template. SULT1A3 insert and vector pET28 were digested with FD BamHI (Thermo Scietific) and FD HindIII (Thermo Scietific) enzymes, then ligated and transformed into *E.coli* Top10 (Invitrogen). Restriction analysis and sequencing were used to confirm the correct sequence gene in the target vector (pET28-His-SULT1A3). Overexpression and purification of sulfotransferase SULT1A3 (~36 kDa) was conducted analogously to AtSOT18. Harvested cells were lysed in a buffer containing: 50 mM HEPES (pH 7.5), 300 mM NaCl, 30 mM imidazole, 5 mM β-merkaptoetanol, 0.1 mg/ml lysozyme, and mixture of protease inhibitors. The lysate was sonicated, centrifuged and loaded on 2 x 5 ml

HisTrap FFTM column (Cytiva) previously equilibrated with a lysis buffer (without lysozyme and protease inhibitors). 6xHis-SULT1A3 protein was eluted with a buffer containing 300 mM imidazole. The final purification step was gel filtration on a Superdex 75 pg HiLoad 26/600 gel filtration column (Cytiva). Fractions with 6xHis-SULT1A3 protein concentrated to 30 μM, flash frozen and stored at -80 °C in a buffer containing 50 mM HEPES (pH 7.5), 100 mM NaCl, 1 mM DTT, 10% glycerol. The homogeneity of both proteins was confirmed by SDS-PAGE electrophoresis (Figure S1).

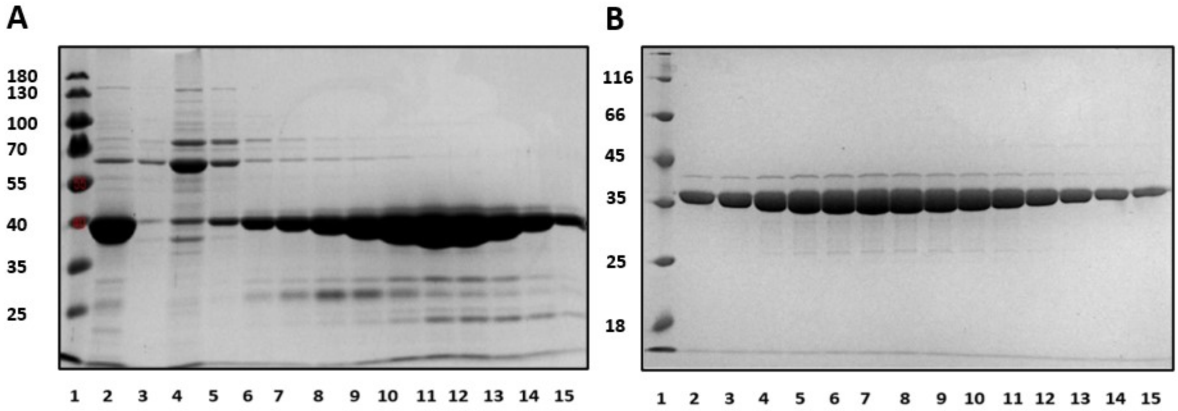

Figure S1. Analysis of protein purity. Purified proteins AtSOT18 (**A**) and SULT1A3 (**B**) were analysed by SDS-PAGE. **A**) Polyacrylamide gel for protein AtSOT18 after gel filtration (Superdex 75 pg 26/600), signal at level ~ 42 kDa, fractions 6-15; **B**) Polyacrylamide gel for protein SULT1A3 after gel filtration (Superdex 75 pg 26/600), signal at level ~ 36 kDa, fraction 2-15 A-PageRuler™ Prestained Protein Ladder (26617), B-Pierce™Unstained Protein MW Marker (26610).

3. Synthesis of 3'-phosphoadenosine 5'-phosphate

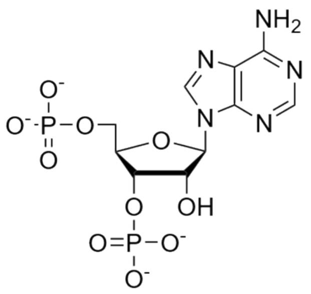

To a solution of adenosine (0.31 g, 1.15 mmol, 1.0 equiv.) in trimethyl phosphate (6.0 mL), $POCl_3$ (0.53 g, 3.45 mmol, 3.0 equiv.) was added at 0 °C. After 1 h, another portion of $POCl_3$ (1.06 g, 6.9 mmol, 6.0 equiv.) was added. The mixture was left overnight at 0 °C and then poured into cold diethyl ether and centrifuged. Supernatant was discarded and the precipitate was diluted in trimethyl phosphate (6.0 mL) followed by addition of 1,2,4-triazole (2.4 g, 34.6 mmol, 30.0 equiv.). After 1 h reaction was quenched with 10 volumes of water and purified by DEAE-Sephadex A-25 column (6.0 × 16.0 cm) using a linear gradient of TEAB (0 – 1.0 M), to obtain 2’,3’-cyclophospoadenosine 5’-phosphate as triethylammonium salt (0.23 mmol, 20% yield), followed by semi-preparative RP-HPLC purification.

Enzymatic cleavage of cPAP (10 mg/mL) was performed with RNAse T2 (750 U, 5 μL/10 mg, MoBiTec) in 50 mM ammonium acetate buffer, pH 7.3.[7] The reaction mixture was placed on termomixer at 37 °C, 300 rpm. Progress of the reaction was monitored by RP HPLC. After 24 h the reaction was purified by 10 K Amicon Ultra-0.5 mL Centrifugal Filters (recovery greater than 90%). As a result, a pure 3’-phosphoadenosine 5’-phosphate was obtained in 17% yield (0.154 g, 0.20 mmol).

2′,3′-cyclophospoadenosine 5′-phosphate (cPAP):

^{1}H NMR (400 MHz, D_2O): δ 8.41 (s, 1H), 8.21 (s, 1H), 6.33 (d, *J* = 4.2 Hz, 1H), 5.44 (ddd, *J* = 10.9, 6.7, 4.2 Hz, 1H), 5.24 − 5.16 (m, 1H), 4.64 − 4.58 (m, 1H), 4.15 − 4.08 (m, 2H).

^{31}P NMR (202 MHz, D_2O): δ 20.70 (dd, *J* = 10.9, 7.4 Hz, 1P), 2.03 (m, 1P).

3′-phosphoadenosine 5′-phosphate (PAP):

^{1}H NMR (400 MHz, D_2O): δ 8.42 (s, 1H), 8.26 (s, 1H), 6.35 (d, *J* = 4.1 Hz, 1H), 5.45 (ddd, *J* = 11.0, 6.7, 4.2 Hz, 1H), 5.21 (td, *J* = 7.2, 3.7 Hz, 1H), 4.66 – 4.60 (m, 1H), 4.15 (t, *J* = 4.3 Hz, 2H).

^{31}P NMR (202 MHz, D_2O): δ 19.79 (dd, *J* = 11.2, 7.5 Hz, 1P), 0.41 – 0.24 (m, 1P).

HRMS: $[M\text{-}H]^-$. calcd for $C_{10}H_{14}N_5O_{10}P_2^-$, 426.02214; found, 426.02268.

4. General Procedure for the Synthesis of PAPP analogues 1–6

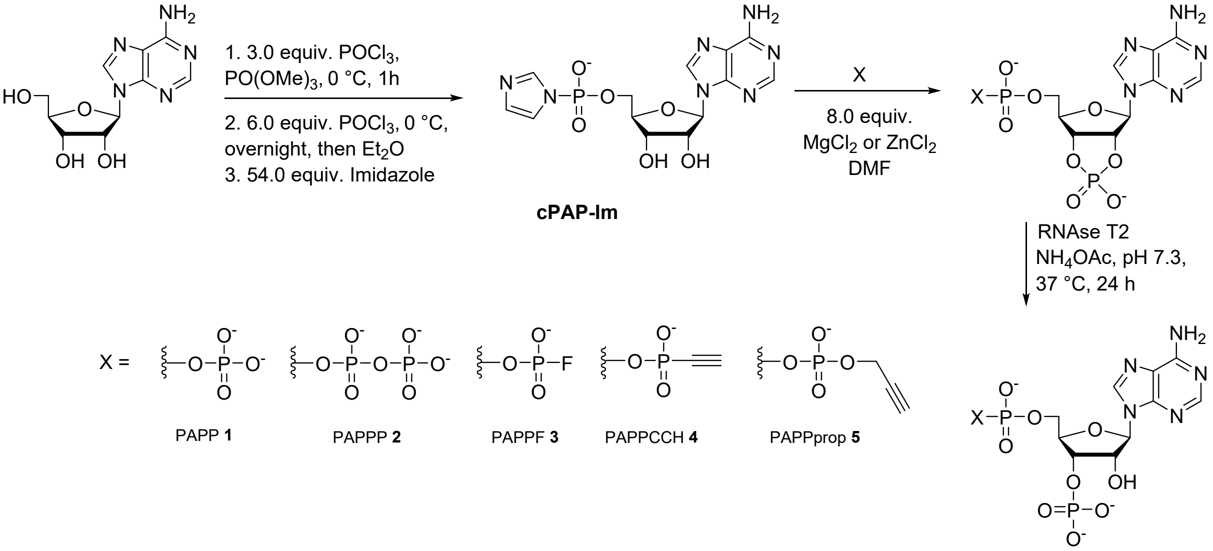

Scheme S1. Synthesis of PAPP analogues **1**–**5**.

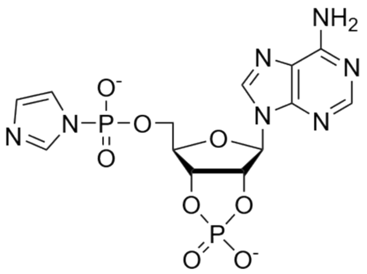

To a solution of adenosine (0.32 g, 1.2 mmol, 1.0 equiv.) in trimethyl phosphate (6.0 mL), $POCl_3$ (0.56 g, 3.64 mmol, 3.0 equiv.) was added at 0 °C. After 1 h, another portion of $POCl_3$ (1.12 g, 7.28 mmol, 6.0 equiv.) was added. The mixture was left overnight at 0 °C and then poured into cold diethyl ether and centrifuged. Supernatant was discarded and the precipitate was diluted in trimethyl phosphate (7.0 mL) followed by addition of imidazole (4.46 g, 65.5 mmol, 54.0 equiv.). After 1 h reaction was quenched with 10 volumes of water and purified by DEAE-Sephadex A-25 column (6.0 × 16.0 cm) using a linear gradient of TEAB (0 – 0.6 M), to obtain 5′-phosphate-2′,3′-cyclophospho adenosine imidazolide as triethylammonium salt (0.22 mmol, 18% yield).

Then, 5′-phosphate-2′,3′-cyclophosphoadenosine imidazolide (cPAP-Im) solution was evaporated under reduced pressure with DMF. Corresponding phosphate and $ZnCl_2$ or $MgCl_2$ (8.0 equiv.) were added. The reaction was stirred until total conversion of starting material observed by RP-HPLC and then quenched by addition of equimolar to $ZnCl_2$ or $MgCl_2$ solution of EDTA in H_2O (the pH was adjusted to 7.0 by the addition of $NaHCO_3$). The resulting cyclic products **1′**–**5′** were purified by DEAE-Sephadex A-25 column using

a linear gradient of TEAB.

Enzymatic cleavage of corresponding cyclic compounds **1′–5′** (10 mg/mL) was performed with RNAse T2 (750 U, 5 μL/10 mg, MoBiTec) in 50 mM ammonium acetate buffer, pH 7.3.[7] The reaction mixture was placed in thermoblock at 37 °C, 300 rpm. The reaction progress was monitored by RP HPLC. After 24 h the reaction was filtered through 10 K Amicon Ultra-0.5 mL Centrifugal Filters. The concentrate was discarded and the flow-through was used for NMR experiments (recovery greater than 90%).

a. 3′-Phosphoadenosine 5′-diphosphate 1

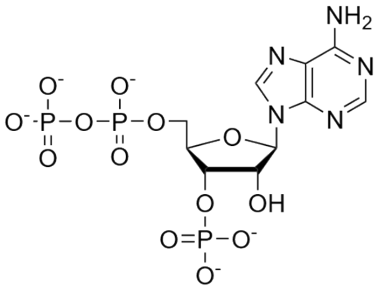

Following the general procedure cPAP-Im solution (0.17 mmol, 1.0 equiv.) was evaporated under reduced pressure with DMF (2.0 ml). Triethylammonium phosphate (0.12 g, 0.50 mmol, 3.0 equiv.) and $ZnCl_2$ (0.18 mg, 1.3 mmol, 8.0 equiv.) were added. The reaction was quenched by adding a 10-fold diluted aqueous EDTA solution (equally molar to $ZnCl_2$), the pH was adjusted to 7.0 with $NaHCO_3$. The resulting product was purified by DEAE-Sephadex A-25 column (4.0 × 15.0 cm) using a linear gradient of TEAB (0 – 1.2 M), to obtain **1′** as triethylammonium salt (0.08 mg, 0.08 mmol, 48%). The cyclic product was then purified on semi-preparative RP-HPLC and subjected to enzymatic cleavage according to the general procedure.

<u>2′,3′-cyclophosphoadenosine 5′-diphosphate **1′**:</u>

^{1}H NMR (500 MHz, D_2O): δ 8.49 (s, 1H), 8.25 (s, 1H), 6.36 (d, *J* = 4.0 Hz, 1H), 5.44 (ddd, *J* = 10.9, 6.7, 4.1 Hz, 1H), 5.30 – 5.23 (m, 1H), 4.69 – 4.64 (m, 1H), 4.28 – 4.23 (m, 2H).

^{31}P NMR (202 MHz, D_2O): δ 20.63 (dd, *J* = 11.1, 7.3 Hz, 1P), -7.86 – -11.13 (m, 2P).

HRMS: $[M-H]^-$. calcd for $C_{10}H_{13}N_5O_{12}P_3^-$, 487.97790; found, 487.97835.

<u>3′-phosphoadenosine 5′-diphosphate **1**:</u>

^{1}H NMR (500 MHz, D_2O): δ 8.57 (s, 1H), 8.27 (s, 1H), 6.18 (d, *J* = 6.7 Hz, 1H), 4.86 – 4.81 (m, overlap with D_2O, 2H), 4.78 – 4.73 (m, overlap with D_2O, 2H), 4.28 – 4.16 (m, 2H), 4.28 – 4.16 (m, 2H).

^{31}P NMR (202 MHz, D_2O): δ 18.59 – 18.19 (m, 1P), 14.80 (td, *J* = 19.9, 10.0 Hz), 0.78 – 0.29 (m, 1P).

HRMS: $[M-H]^-$. calcd for $C_{10}H_{15}N_5O_{13}P_3^-$, 505.98847; found, 505.98894.

b. 3′-Phosphoadenosine 5′-triphosphate 2

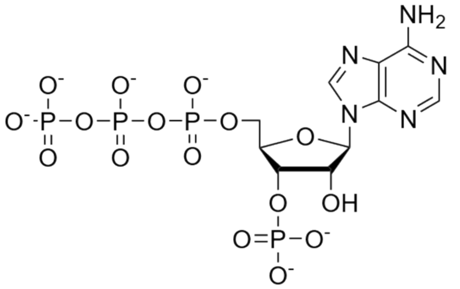

Following the general procedure cPAP-Im solution (0.13 mmol, 1.0 equiv.) was evaporated under reduced pressure with DMF (2.0 ml). Triethylammonium pyrophosphate (0.15 g, 0.39 mmol, 3.0 equiv.) and $ZnCl_2$ (0.14 g, 1.0 mmol, 8.0 equiv.) were added. After 20 h reaction was quenched by adding a 10-fold diluted aqueous EDTA solution, the pH was adjusted to 7.0 with $NaHCO_3$. The resulting product was purified by DEAE-Sephadex A-25 column (4.0 × 15.0 cm) using a linear gradient of TEAB (0 – 1.2 M), to obtain **2′** as triethylammonium salt (0.08 mmol, 63% yield). The cyclic product was then purified on semi-preparative RP-HPLC (38 mg, 0.05 mmol, 39%) and subjected to enzymatic cleavage according to the general procedure.

2′,3′-cyclophosphoadenosine 5′-triphosphate **2′**:

^{1}H NMR (500 MHz, D_2O): δ 8.38 (s, 1H), 8.15 (s, 1H), 6.29 (d, *J* = 4.3 Hz, 1H), 5.43 (ddd, *J* = 4.3, 6.7, 11.0 Hz, 1H), 5.27 − 5.22 (m, 1H), 4.65 − 4.59 (m, 1H), 4.31 − 4.25 (m, 2H).

^{31}P NMR (202 MHz, D_2O): δ 20.76 (dd, *J* = 7.5, 11.0 Hz, 1P), -7.07 (d, *J* = 19.7 Hz, 1P), -10.40 (d, *J* = 19.3, 5.7 Hz, 1P), -21.33 (dd, *J* = 19.7, 19.3 Hz, 1P).

HRMS: $[M-H]^-$. calcd for $C_{10}H_{14}N_5O_{15}P_4^-$, 567.94423; found, 567.94488.

3′-phospoadenosine 5′-triphosphate **2**:

^{1}H NMR (500 MHz, D_2O): δ 8.58 (s, 1H), 8.27 (s, 1H), 6.18 (d, *J* = 5.9 Hz, 1H), 4.90 − 4.85 (m, 1H), 4.82 − 4.78 (m, overlap with D_2O, 1H), 4.64 − 4.58 (m, 1H), 4.34 − 4.22 (m, 2H), 4.31 − 4.25 (m, 2H).

^{31}P NMR (202 MHz, D_2O): δ 1.09 − 0.92 (m, 1P), -8.63 (d, *J* = 17.3 Hz, 1P), -10.27 (d, *J* = 18.6, 1P), -21.00 − -21.37 (m, 1P).

HRMS: $[M-H]^-$. calcd for $C_{10}H_{16}N_5O_{16}P_4^-$, 585.95480; found, 585.95546.

c. 3′-Phosphoadenosine 5′-fluoro diphosphate 3

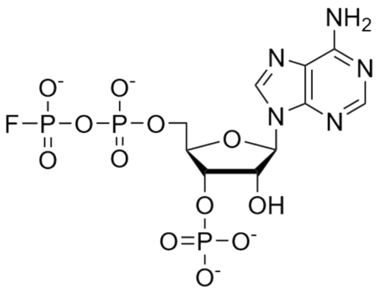

Following the general procedure cPAP-Im solution (0.17 mmol, 1.0 equiv.) was evaporated under reduced pressure with DMF (2.0 ml). Triethylammonium fluorophosphate salt (0.13 g, 0.50 mmol, 3.0 equiv.) and $ZnCl_2$ (0.18 g, 1.34 mmol, 8.0 equiv.) were added. After 18 h reaction was quenched by adding a 10-fold diluted aqueous EDTA solution, the pH was adjusted to 7.0 with $NaHCO_3$. The resulting product was purified by DEAE-Sephadex A-25 column (4.0 × 15.0 cm) using a linear gradient of TEAB (0 – 1.0 M), to obtain **3′** as triethylammonium salt (0.10 mmol, 60% yield). The cyclic product was then purified on semi-preparative RP-HPLC (38 mg, 0.05 mmol, 39%) and subjected to enzymatic cleavage according to the general procedure.

<u>2′,3′-cyclophosphoadenosine 5′-fluoro diphosphate **3′**:</u>

^{1}H NMR (400 MHz, D_2O): δ 8.42 (s, 1H), 8.28 (s, 1H), 6.37 (d, *J* = 4.1 Hz, 1H), 5.50 − 5.42 (m, 1H), 5.28 − 5.21 (m, 1H), 4.68 − 4.64 (m, 1H), 4.30 − 4.22 (m, 2H).

^{31}P NMR (162 MHz, D_2O): δ 20.62 (dd, *J* = 7.4, 11.0 Hz, 1P), -10.65 − -10.92 (m, 1P), -17.04 (dd, *J* = 19.7, 934.4 Hz, 1P).

HRMS: $[M-H]^-$. calcd for $C_{10}H_{12}FN_5O_{11}P_3^-$, 489.97357; found, 489.97393.

<u>3′-phosphoadenosine 5′-fluoro diphosphate **3**:</u>

^{1}H NMR (500 MHz, D_2O): δ 8.55 (s, 1H), 8.32 (s, 1H), 6.21 (d, *J* = 6.6 Hz, 1H), 4.91 − 4.82 (m, 2H), 4.64 − 4.59 (m, 1H), 4.28 − 4.23 (m, 2H).

^{31}P NMR (202 MHz, D_2O): δ 1.17 - 0.48 (m, 1P), -10.39 − -10.74 (m, 1P), -14.45 − -19.43 (m, 1P).

^{19}F NMR (471 MHz, D_2O): δ - 73.61 (d, *J* = 934 Hz, 1F).

HRMS: $[M-H]^-$. calcd for $C_{10}H_{14}FN_5O_{12}P_3^-$, 507.98413; found, 507.98449.

d. 3′-Phosphoadenosine 5′-phosphate-C-ethynyl phosphonate 4

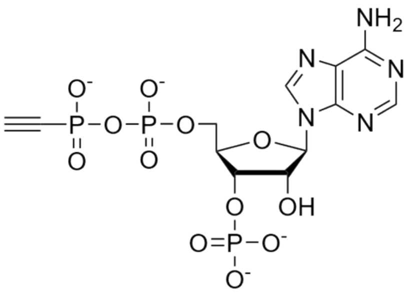

Following the general procedure cPAP-Im solution (0.14 mmol, 1.0 equiv.) was evaporated under reduced pressure with DMF (1.0 ml). Triethylammonium salt of 1-ethynyl-phosphonate (12 mg, 0.56 mmol, 4.0 equiv.) dissolved in DMF (1.0 ml) and $MgCl_2$ (54 mg, 0.56 mmol, 4.0 equiv.) were added. After 24 h reaction was quenched by adding a 10-fold diluted aqueous EDTA solution, the pH was adjusted to 7.0 with $NaHCO_3$. The resulting product was purified by DEAE-Sephadex A-25 column (4.0 × 15.0 cm) using a linear gradient of TEAB (0 – 1.0 M), to obtain **4′** as triethylammonium salt (0.06 mmol, 36% yield). The cyclic product was then purified on semi-preparative RP-HPLC and subjected to enzymatic cleavage according to the general procedure.

2′,3′-cyclophosphoadenosine 5′-phosphate-C-ethynyl phosphonate **4′**:

^{1}H NMR (500 MHz, D_2O): δ 8.44 (s, 1H), 8.27 (s, 1H), 6.37 (d, *J* = 4.2 Hz, 1H), 5.46 (ddd, *J* = 11.1, 6.7, 4.1 Hz, 1H), 5.30 − 5.24 (m, 1H), 4.69 − 4.64 (m, 1H), 4.31 − 4.24 (m, 2H), 3.14 (d, *J* = 12.9 Hz, 1H).

^{31}P NMR (202 MHz, D_2O): δ 20.67 (dd, *J* = 11.2, 7.4 Hz, 1P), -11.08 − -11.35 (m, 1P), -20.98 (dd, *J* = 22.2, 12.4 Hz, 1P).

HRMS: $[M-H]^-$. calcd for $C_{12}H_{13}N_5O_{11}P_3^-$, 495.98299; found, 495.98262.

3′-phosphoadenosine 5′-phosphate-C-ethynyl phosphonate **4**:

^{1}H NMR (500 MHz, D_2O): δ 8.55 (s, 1H), 8.27 (s, 1H), 6.19 (d, *J* = 7.0 Hz, 1H), 4.88 – 4.84 (m, 1H), 4.84 – 4.80 (m, overlap with D_2O, 2H), 4.76 – 4.73 (m, overlap with D_2O, 1H), 4.63 – 4.58 (m, 1H), 4.30 – 4.20 (m, 2H), 3.15 (d, *J* = 12.8 Hz, 1H).

^{31}P NMR (202 MHz, D_2O): δ 1.82 (s, 1P), -11.71 – -12.23 (m, 1P), -21.90 (dd, *J* = 21.8, 13.1 Hz, 1P).

HRMS: $[M-H]^-$. calcd for $C_{12}H_{15}N_5O_{12}P_3^-$, 513.99355; found, 513.99379.

e. 3′-Phosphoadenosine 5′-propargyl diphosphate 5

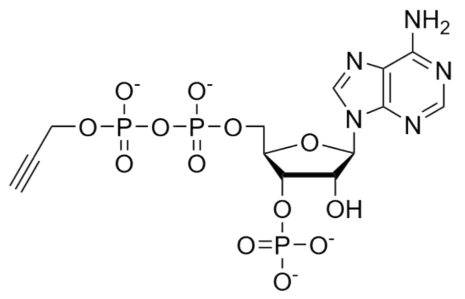

Following the general procedure cPAP-Im solution (0.06 mmol, 1.0 equiv.) was evaporated under reduced pressure with DMF (1.0 ml). Triethylammonium salt of propargyl phosphate (42 mg, 0.18 mmol, 3.0 equiv.) and $MgCl_2$ (45 mg, 0.47 mmol, 8.0 equiv.) were added. After 20 h reaction was quenched by adding a 10-fold diluted aqueous EDTA solution, the pH was adjusted to 7.0 with $NaHCO_3$. The resulting product was purified by DEAE-Sephadex A-25 column (3.0 × 7.0 cm) using a linear gradient of TEAB (0 – 1.0 M), to obtain **5′** as triethylammonium salt (0.03 mmol, 52% yield). The cyclic product was then purified on semi-preparative RP-HPLC and subjected to enzymatic cleavage according to the general procedure.

<u>2′,3′-cyclophosphoadenosine 5′-propargyl diphosphate **5′**:</u>

^{1}H NMR (400 MHz, D_2O): δ 8.43 (s, 1H), 8.25 (s, 1H), 6.37 (d, *J* = 4.1 Hz, 1H), 5.51 − 5.44 (m, 1H), 5.29 − 5.23 (m, 1H), 4.66 − 4.61 (m, 1H), 4.49 − 4.44 (m, 2H), 4.28 − 4.23 (m, 2H), 2.79 (t, *J* = 2.4 Hz, 1H).

^{31}P NMR (162 MHz, D_2O): δ 20.70 (dd, *J* = 10.8, 7.7 Hz, 1P), -10.53 − -10.79 (m, 2P).

HRMS: $[M-H]^-$. calcd for $C_{13}H_{15}N_5O_{12}P_3^-$, 525.99355; found, 525.99406.

<u>3'-phosphoadenosine 5′-propargyl diphosphate **5**:</u>

^{1}H NMR (400 MHz, D_2O): δ 8.55 (s, 1H), 8.27 (s, 1H), 6.18 (d, *J* = 7.0 Hz, 1H), 4.90 – 4.84 (m, 1H), 4.85 – 4.80 (m, overlap with D_2O, 2H), 4.77 – 4.72 (m, overlap with D_2O, 1H), 4.59 (s, 1H), 4.52 – 4.47 (m, 2H), 4.23 (s, 2H), 2.80 – 2.76 (m, 1H).

^{31}P NMR (162 MHz, D_2O): δ ^{31}P NMR (202 MHz, D_2O) δ 1.57 (s, 1P), 0.49 (s, 1P), -11.48 (m, 1P).

HRMS: $[M-H]^-$. calcd for $C_{13}H_{17}N_5O_{13}P_3^-$, 544.00412; found, 544.00474.

5. Synthesis of 3′-phosphoadenosine 5′-methylene bis-phosphonate 6

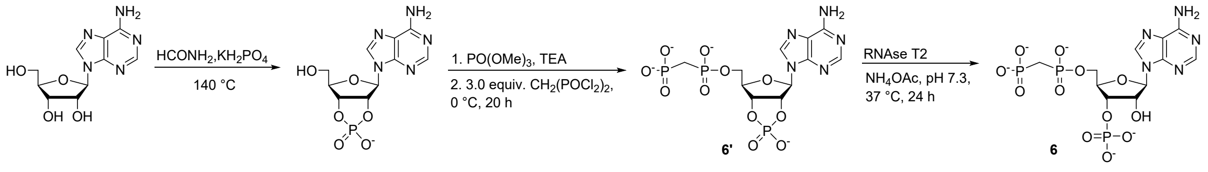

Scheme S2. Synthesis of 3'-phosphoadenosine 5'-methylene bis-phosphonate **6**.[8]

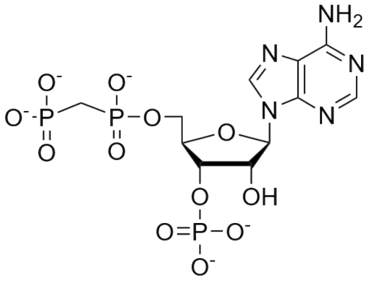

Adenosine (0.21 g, 0.8 mmol) was dissolved in formamide (40.0 ml), KH_2PO_4 (0.1M) was added. The reaction was carried out at 140 °C for 12 h and then at room temperature for 12 h. The resulting product was purified by DEAE-Sephadex A-25 column (6.0 × 16.0 cm) using a linear gradient of TEAB (0 – 0.6 M), to obtain 2′,3′-cyclophosphoadenosine as triethylammonium salt (0.33 mmol, 28% yield).

To a solution of 2′,3′-cyclophospho adenosine (0.12 g, 0.29 mmol), 2.5 μl of TEA was added in trimethyl phosphate (2.5 mL) at 0 °C. Next, methyl bis-dichlorophosphate (0.88 mmol, 3.0 equiv.) was added. After 20 h, 0.7 M aqueous TEAB was added to pH 7. The resulting product was purified by DEAE-Sephadex A-25 column (6.0 × 16.0 cm) using a linear gradient of TEAB (0 – 0.5 M), to obtain **6′** as triethylammonium salt (0.015 mmol, 6% yield). The cyclic product was then purified on semi-preparative RP-HPLC and subjected to enzymatic cleavage according to the general procedure.

2′,3′-cyclophosphoadenosine 5′-methylene bis-phosphonate **6′**:

^{1}H NMR (500 MHz, D$_2$O): δ 8.60 (s, 1H), 8.40 (s, 1H), 6.41 (d, *J* = 3.7 Hz, 1H), 5.50 (ddd, *J* = 10.4, 6.6, 3.7 Hz, 1H), 5.32 − 5.25 (m, 1H), 4.70 − 4.66 (m, 1H), 4.27 − 4.17 (m, 2H), 2.18 – 2.06 (m, 2H).

^{31}P NMR (202 MHz, D$_2$O): δ 20.59 (dd, *J* = 10.7, 8.2 Hz, 1P), 19.22 − 18.81 (m, 1P), 16.45 − 15.95 (m, 1P).

HRMS: [M-H]$^-$. calcd for $C_{11}H_{15}N_5O_{11}P_3^-$, 485.99864; found, 485.99932.

3′-phosphoadenosine 5′-methylene bis-phosphonate **6**:

^{1}H NMR (500 MHz, D$_2$O): δ 8.58 (s, 1H), 8.28 (s, 1H), 6.18 (d, *J* = 6.3 Hz, 1H), 4.92 – 4.88 (m,

1H), 4.88 – 4.82 (m, 2H), 4.76 – 4.73 (m, overlap with D_2O, 1H), 4.59 – 4.55 (m, 1H), 4.21 – 4.15 (m, 2H), 2.24 – 2.13 (m, 2H).

^{31}P NMR (202 MHz, D_2O): δ 8.70 – 18.16 (m, 1P), 14.80 (td, *J* = 19.9, 10.0 Hz), 0.49 (m, 1P).

HRMS: $[M-H]^-$. calcd for $C_{11}H_{17}N_5O_{12}P_3^-$, 504.00920; found, 504.00951.

6. General Procedure for the Synthesis of C8-modified analogues 7,8

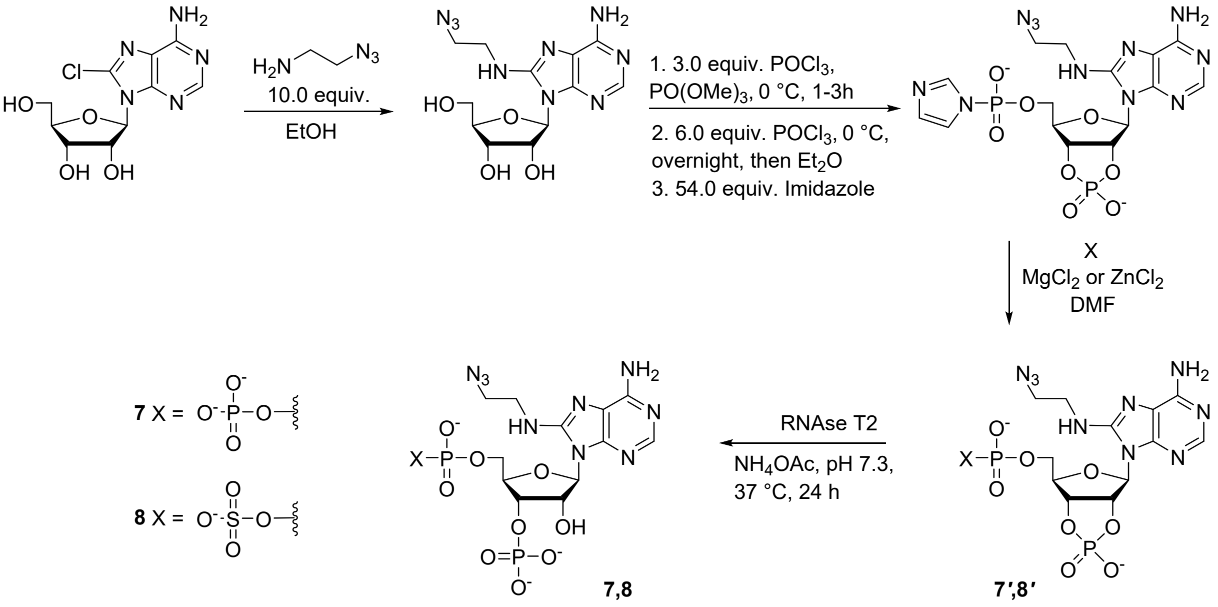

Scheme S3. Synthesis of C8-(1-amino-2-azidoethyl) analogs **7**,**8**.

To a solution of C8-chloroadenosine (0.12 g, 0.35 mmol, 1.0 equiv.) in ethanol (2.0 ml), 1-amino-2-azido ethyl (0.30 g, 3.5 mmol, 10 equiv.) was added and the reaction mixture was stirred under microwave irradiation for 1.5 h at 160 °C, 50W. The progress of the reaction was analyzed by RP HPLC. The product was purified by gel chromatography (DCM:MeOH, 9:1) to obtain C8-(1-amino-2-azidoethyl) adenosine in 64% yield (0.22 mmol). Further reaction steps were carried out according to the procedure for obtaining PAPP (4. General Procedure for the Synthesis of PAPP analogues) or PAPS analogues (13. General Procedure for the Synthesis of Fluorinated PAPS).

a. 3′-Phospho-8-(1-amino-2-azidoethyl) adenosine 5′-phosphate 7

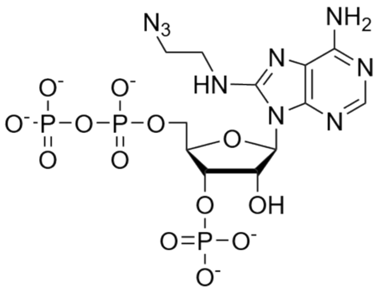

Following the general procedure, C8-(1-amino-2-azido-ethyl) adenosine (86 mg, 0.15 mmol, 1.0 equiv.), $POCl_3$ (68 mg, 0.44 mmol, 3.0 equiv.) in trimethyl phosphate (1.0 mL) were used at 0 °C. After 3 h, the second portion of $POCl_3$ (136 mg, 0.89 mmol, 6.0 equiv.) was added. The mixture was left overnight at 0 °C, then poured into cold diethyl ether and centrifuged. Supernatant was discarded and the precipitate was diluted in trimethyl phosphate (2.0 mL) followed by addition of imidazole (543 mg, 8.0 mmol, 54.0 equiv.). After 1 h the reaction was quenched with 10 volumes of water and purified by DEAE-Sephadex A-25 column (4.0 × 15.0 cm) a linear gradient of TEAB (0 – 0.8 M), to obtain the product as triethylammonium salt (0.033 mmol, 22% yield).

Next, C8-AE-cPAP-Im (0.012 mmol, 1.0 equiv.), triethylamine phosphate (9 mg, 0.035 mmol, 2.0 equiv.) and $ZnCl_2$ (15 mg, 0.1 mmol, 8.0 equiv.) were mixed in DMF (2.0 mL). After 24 h, cyclic 2’,3’-phospho-8-(1-amino-2-azidoethyl) adenosine 5’-phosphate **7′** was purified by DEAE-Sephadex A-25 column (3.0 × 7.0 cm) using a linear gradient of TEAB (0 – 0.6 M) (10.0 µmol, 84% yield), followed by semi-preparative RP HPLC (5.0 µmol, 43% yield) and subjected to enzymatic cleavage according to the general procedure.

2′,3′-cyclophospho-8-(1-amino-2-azido ethyl) adenosine 5′-phosphate **7′**:

^{1}H NMR (500 MHz, D_2O): δ 8.16 (s, 1H), 6.20 (d, *J* = 4.6 Hz, 1H), 5.45 – 5.38 (m, 1H), 5.29 – 5.21 (m, 1H), 4.59 – 4.51 (m, 1H), 4.40 – 4.27 (m, 2H), 3.75 (t, *J* = 6.0 Hz, 2H), 3.69 – 3.60 (m, 2H).

^{31}P NMR (202 MHz, D_2O): δ 20.28 (t, *J* = 9.3 Hz, 1P), -10.63 (d, *J* = 21.1 Hz, 1P), -11.44 – -11.67 (m, 1P).

HRMS: $[M-H]^-$. calcd for $C_{12}H_{17}N_9O_{12}P_3^-$, 572.02150; found, 572.02205.

3′-phospho-8-(1-amino-2-azido ethyl) adenosine 5′-phosphate **7**:

^{1}H NMR (500 MHz, D_2O): δ 8.20 (s, 1H), 6.12 (d, *J* = 7.8 Hz, 1H), 4.91 – 4.86 (m, 1H), 4.84 (s, 1H), 4.60 – 4.55 (m, 1H), 4.34 – 4.20 (m, 3H), 3.81 – 3.71 (m, 2H), 3.68 – 3.54 (m, 3H).

^{31}P NMR (202 MHz, D_2O): δ -0.07 (d, *J* = 8.7 Hz, 1P), -10.85 (d, *J* = 19.9 Hz, 1P), -11.74 – -12.04 (m, 1P).

HRMS: $[M-H]^-$. calcd for $C_{12}H_{19}N_9O_{13}P_3^-$, 590.03206; found, 590.03280.

b. 3′-Phospho-8-(1-amino-2-azidoethyl) adenosine 5′-phosphosulfate 8

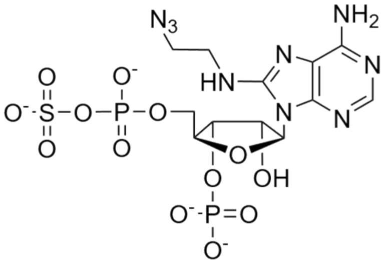

Following the general procedure, C8-(1-amino-2-azido-ethyl) adenosine (86 mg, 0.15 mmol, 1.0 equiv.), $POCl_3$ (68 mg, 0.44 mmol, 3.0 equiv.) in trimethyl phosphate (1.0 mL) were used at 0 °C. After 3 h, the second portion of $POCl_3$ (136 mg, 0.89 mmol, 6.0 equiv.) was added. The mixture was left overnight at 0 °C, then poured into cold diethyl ether and centrifuged. Supernatant was discarded and the precipitate was diluted in trimethyl phosphate (2.0 mL) followed by addition of imidazole (543 mg, 8.0 mmol, 54.0 equiv.). After 1 h the reaction was quenched with 10 volumes of water and purified by DEAE-Sephadex A-25 column (4.0 × 15.0 cm) using a linear gradient of TEAB (0 – 0.8 M), to obtain the product as triethylammonium salt (0.033 mmol, 22% yield). Next, C8-AE-cPAP-Im (0.03 mmol, 1.0 equiv.), bis(tributylammonium) sulfate (33 mg, 0.07 mmol, 2.0 equiv.) and $MgCl_2$ (28 mg, 0.29 mmol, 9.0 equiv.) were mixed in DMF (2.0 mL). After 24 h, cyclic 2′,3′-phospho-C8-(1-amino-2-azidoethyl) adenosine 5′-phosphosulfate **8′** was purified by DEAE-Sephadex A-25 column (3.0 × 7.0 cm) using a linear gradient of TEAB (0 – 1.0 M) (0.013 mmol, 43% yield), followed by semi-preparative RP HPLC and subjected to enzymatic cleavage according to the general procedure.

<u>5′-phosphosulphate-2′,3′-cyclophospho-C8-(1-amino-2-azidoethyl) adenosine **8′**:</u>

MS-ESI: $[M-H]^-$. calcd for $C_{12}H_{16}N_9O_{12}P_2S^-$, 572.0; found, 572.9.

<u>5′-phosphosulfate -3′-phospho-C8-(1-amino-2-azidoethyl) adenosine **8**:</u>

^{1}H NMR (500 MHz, D_2O): δ 8.19 (s, 1H), 6.13 (d, *J* = 8.1 Hz, 1H), 4.90 – 4.84 (m, 1H), 4.85 – 4.81 (m, 1H), 4.61 – 4.56 (m, 1H), 4.37 – 4.23 (m, 3H), 3.77 (q, *J* = 5.3 Hz, 2H), 3.68 – 3.54 (m, 3H).

^{31}P NMR (202 MHz, D_2O): δ -0.09 (d, *J* = 7.5 Hz, 1P), -11.11 – -11.32 (m, 1P).

MS-ESI: $[M-H]^-$. calcd for $C_{12}H_{18}N_9O_{13}P_2S^-$, 590.02255; found, 590.02040.

7. Synthesis of 3′-phospho-6-(1-amino-2-azidoethyl) adenosine 5′-diphosphate 9

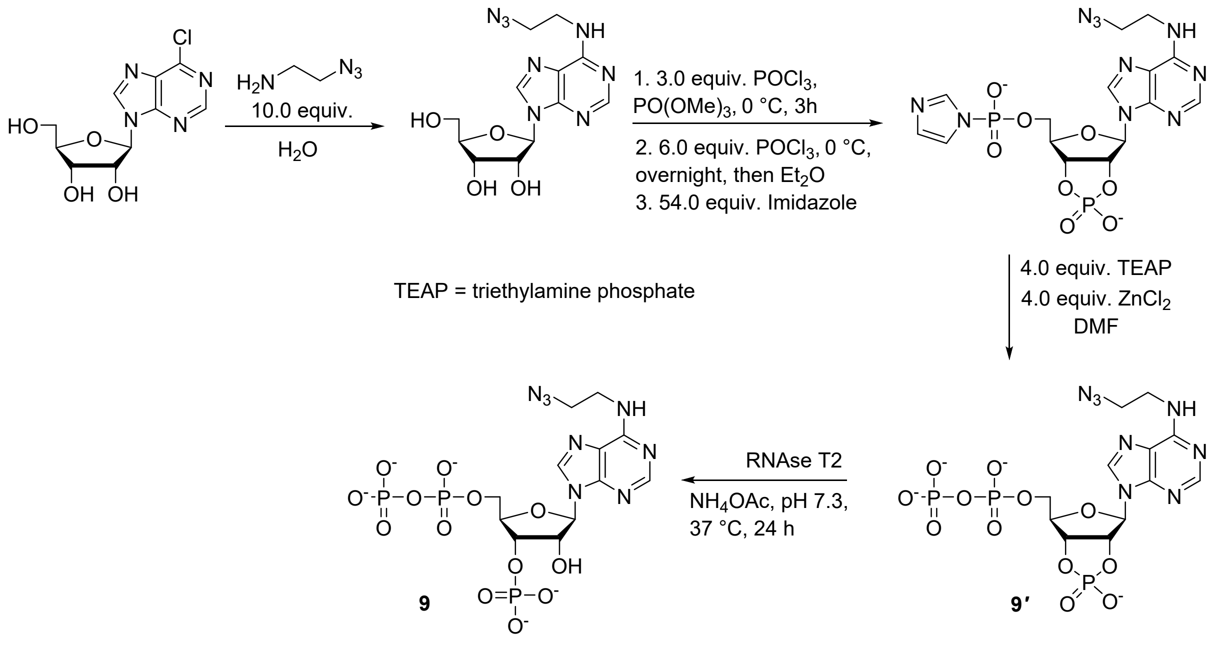

Scheme S4. Synthesis of N6-(1-amino-2-azidoethyl) analog **9**.

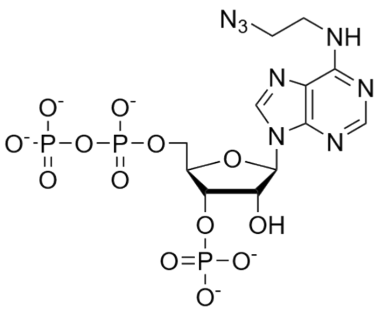

To a solution of 6-chloropurine riboside (1.0 equiv.) in H_2O (2.0 ml), 1-amino-2-azido ethyl (10.0 equiv.) was added at 0 °C. The reaction was carried out for 1 h. The progress of the reaction was monitored by RP HPLC. The product was purified by gel chromatography (DCM:MeOH, 9:1). Next, following the general procedure, 6-(1-amino-2-azidoethyl)adenosine (0.1 g, 0.3 mmol, 1.0 equiv.), $POCl_3$ (0.14 g, 0.9 mmol, 3.0 equiv.) in trimethyl phosphate (2.0 mL) were used at 0 °C. After 3 h, the second portion of $POCl_3$ (0.28 g, 1.8 mmol, 6.0 equiv.) was added. The mixture was left overnight at 0 °C, then poured into cold diethyl ether and centrifuged. Supernatant was discarded and the precipitate was diluted in trimethyl phosphate (2.0 mL) followed by addition of imidazole (1.1 g, 16.2 mmol, 54.0 equiv.). After 1 h the reaction was quenched with 10 volumes of water and purified by DEAE-Sephadex A-25 column (6.0 × 16.0 cm) using a linear gradient of TEAB (0 – 0.8 M), to obtain the product as triethylammonium salt (0.1 mmol, 34% yield). Next, N6-AE-cPAP-Im (0.07 mmol, 1.0 equiv.), triethylamine phosphate (58 mg, 0.3 mmol, 4.0 equiv.) and $ZnCl_2$ (40 mg, 0.3 mmol, 4.0 equiv.) were mixed in DMF (2.0 mL). Cyclic 2′,3′-phospho-6-(1-amino-2-azidoethyl) adenosine 5′-

diphosphate **9´** was purified by DEAE-Sephadex A-25 column (4.0 × 15.0 cm) using a linear gradient of TEAB (0 – 1.0 M) (23.0 mg, 0.03 mmol, 41% yield), followed by semi-preparative RP HPLC and subjected to enzymatic cleavage according to the general procedure.

2′,3′-cyclophospho-6-(1-amino-2-azido ethyl) adenosine 5′-diphosphate **9´**:

^{1}H NMR (500 MHz, D_2O): δ 8.46 (s, 1H), 8.33 (s, 1H), 6.38 (d, *J* = 4.2 Hz, 1H), 5.46 (ddd, *J* = 11.5, 6.6, 4.2 Hz, 1H), 5.30 – 5.23 (m, 1H), 4.69 – 4.64 (m, 1H), 4.26 (dd, *J* = 5.2, 3.4 Hz, 2H), 3.85 (bs, 2H), 3.65 – 3.59 (m, 2H).

^{31}P NMR (202 MHz, D_2O): δ 19.71 (dd, *J* = 11.5, 7.0 Hz, 1P), -10.79 (d, *J* = 20.9 Hz, 1P), -11.51 (d, *J* = 20.9 Hz, 1P).

HRMS: $[M-H]^-$. calcd for $C_{12}H_{16}N_8O_{12}P_3^-$, 557.01060; found, 557.00977.

3′-phospho-6-(1-amino-2-azido ethyl) adenosine 5′-diphosphate **9**:

^{1}H NMR (500 MHz, D_2O): δ 8.52 (s, 1H), 8.30 (s, 1H), 6.18 (d, *J* = 6.1 Hz, 1H), 4.90 – 4.82 (m, 2H), 4.62 – 4.55 (m, 1H), 4.31 – 4.16 (m, 2H), 3.84 (s, 2H), 3.64 (t, *J* = 5.7 Hz, 2H).

^{31}P NMR (202 MHz, D_2O): δ 1.45 (s, 1P), -9.72 – -10.11 (m, 1P), -11.15 (d, *J* = 21.1 Hz, 1P).

HRMS: $[M-H]^-$. calcd for $C_{12}H_{18}N_8O_{13}P_3^-$, 575.02117; found, 575.02126.

8. General Procedure for the Synthesis of N6-modified analogues 10,11

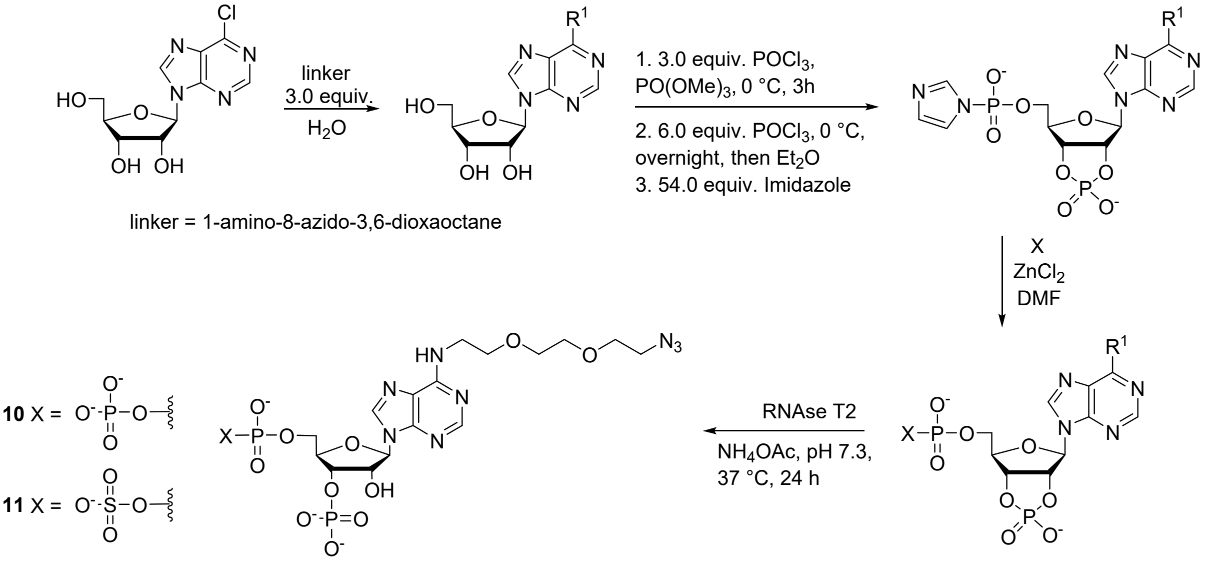

10,11 **10′,11′**

Scheme S5. Synthesis of N6-(1-amino-8-azido-3,6-dioxyoctano) analogs **10**,**11**.

To a solution of 6-chloropurine riboside (0.3 g, 1.0 mmol, 1.0 equiv.) in water (2.0 ml), 1-amino-8-azido-3,6-dioxaoctane (0.52 g, 3.0 mmol, 3.0 equiv.) was added, and the reaction mixture was stirred for 2 h at room temperature. The progress of the reaction was analyzed by RP HPLC. The product was purified by gel chromatography (DCM: MeOH, 9:1) to obtain N6-(1-amino-8-azido-3,6-dioxyoctano) adenosine in 81% yield (0.84 mmol). Further reaction steps were carried out according to the procedure for obtaining PAPP (4. General Procedure for the Synthesis of PAPP analogues) or PAPS analogues (13. General Procedure for the Synthesis of Fluorinated PAPS).

a. 3′-Phospho-6-(8-azido-3,6-dioxyoctano-1-amino) adenosine 5′-diphosphate 10

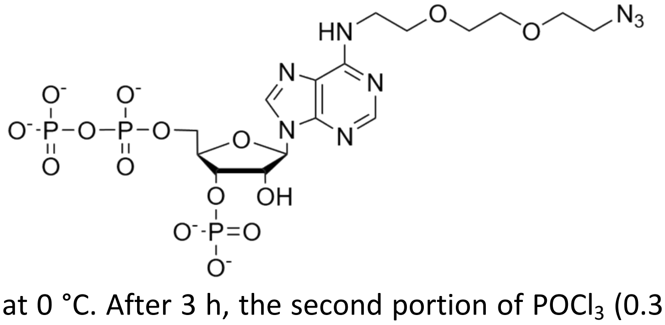

Following the general procedure, N6-(1-amino-8-azido-3,6-dioxyoctano) adenosine (0.15 g, 0.4 mmol, 1.0 equiv.), $POCl_3$ (0.16 g, 1.2 mmol, 3.0 equiv.) in trimethyl phosphate (1.0 mL) were used at 0 °C. After 3 h, the second portion of $POCl_3$ (0.32 g, 2.4 mmol, 6.0 equiv.) was added. The

mixture was left overnight at 0 °C, then poured into cold diethyl ether and centrifuged. Supernatant was discarded and the precipitate was diluted in trimethyl phosphate (2.0 mL) followed by addition of imidazole (1.29 mg, 18.9 mmol, 54.0 equiv.). After 1 h the reaction was quenched with 10 volumes of water and purified by DEAE-Sephadex A-25 column (6.0 × 16.0 cm) using a linear gradient of TEAB (0 – 0.8 M), to obtain product as triethylammonium salt (0.12 mmol, 35% yield).

Next, obtained N6-PEG-cPAP-Im (0.12 mmol, 1.0 equiv.), triethylamine phosphate (97 mg, 0.48 mmol, 4.0 equiv.) and $ZnCl_2$ (66 mg, 0.48 mmol, 4.0 equiv.) were mixed in DMF (2.0 mL). After 24 h, cyclic 2’,3’-phospho-6-(8-azido-3,6-dioxyoctano-1-amino) adenosine 5’-diphosphate **10′** was purified by DEAE-Sephadex A-25 column (4.0 × 15.0 cm) using a linear gradient of TEAB (0 – 1.0 M) (38 mg, 0.04 mmol, 37% yield), followed by semi-preparative RP HPLC and subjected to enzymatic cleavage according to the general procedure.

2′,3′-cyclophospho N6-(8-azido-3,6-dioxyoctano-1-amino) adenosine 5′-diphosphate **10′**:

^{1}H NMR (500 MHz, D_2O): δ 8.47 (s, 1H), 8.33 (s, 1H), 6.38 (d, *J* = 4.2 Hz, 1H), 5.46 (ddd, *J* = 11.1, 6.6, 4.2 Hz, 1H), 5.30 – 5.23 (m, 1H), 4.69 – 4.64 (m, 1H), 4.25 (dd, *J* = 5.2, 3.4 Hz, 2H), 3.85 (s, 4H), 3.76 – 3.66 (m, 4H), 3.66 – 3.60 (m, 2H), 3.41 – 3.35 (m, 2H).

^{31}P NMR (202 MHz, D_2O): δ 19.69 (dd, *J* = 11.5, 7.0 Hz, 1P), -10.80 (d, *J* = 20.9 Hz, 1P), -11.53 (d, *J* = 20.9 Hz, 1P).

HRMS: $[M-H]^-$. calcd for $C_{16}H_{24}N_8O_{14}P_3^-$, 645.06303; found, 645.06232.

3′-phospho N6-(8-azido-3,6-dioxyoctano-1-amino) adenosine 5′-diphosphate **10**:

^{1}H NMR (500 MHz, D_2O): δ 8.51 (s, 1H), 8.27 (s, 1H), 6.16 (d, *J* = 6.3 Hz, 1H), 4.86 – 4.82 (m, 3H), 4.60 – 4.55 (m, 1H), 4.28 – 4.17 (m, 2H), 3.80 (s, 4H), 3.79 – 3.75 (m, 2H), 3.74 – 3.66 (m, 4H), 3.37 (t, *J* = 4.9 Hz, 2H).

^{31}P NMR (202 MHz, D_2O): δ 1.33 (d, *J* = 8.7 Hz, 1P), -9.96 (d, *J* = 19.9 Hz, 1P), -11.20 (d, *J* = 19.9 Hz, 1P).

HRMS: $[M-H]^-$. calcd for $C_{16}H_{26}N_8O_{15}P_3^-$, 663.07360; found, 663.07353.

b. 3′-Phospho-N6-(1-amino-8-azido-3,6-dioxyoctano) adenosine 5′-phosphosulfate 11

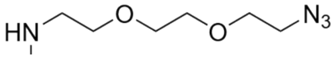

Following the general procedure, N6-(1-amino-8-azido-3,6-dioxyoctano) adenosine (0.1 g, 0.26 mmol, 1.0 equiv.), $POCl_3$ (0.12 g, 0.78 mmol, 3.0 equiv.) in trimethyl phosphate (1.0 mL) were used at 0 °C. After 3 h, the second portion of $POCl_3$ (0.24 g, 1.56 mmol, 6.0 equiv.) was added. The mixture was left overnight at 0 °C, then poured into cold diethyl ether and centrifuged. Supernatant was discarded and the precipitate was diluted in trimethyl phosphate (2.0 mL) followed by addition of imidazole (0.95 g, 14.0 mmol, 54.0 equiv.). After 1 h the reaction was quenched with 10 volumes of water and purified by DEAE-Sephadex A-25 column (6.0 × 16.0 cm) using a linear gradient of TEAB (0 – 0.8 M), to obtain product as triethylammonium salt (0.08 mmol, 53% yield).

Next, obtained N6-PEG-cPAP-Im (0.04 mmol, 1.0 equiv.), bis(tributylammonium) sulfate (39 mg, 0.08 mmol, 2.0 equiv.) and $MgCl_2$ (33 mg, 0.35 mmol, 9.0 equiv.) were mixed in DMF (2.0 mL). After 24 h, cyclic 2′,3′-phospho-N6-(1-amino-8-azido-3,6-dioxyoctano) adenosine 5′-phosphosulfate **11′** was purified by DEAE-Sephadex A-25 column (4.0 × 15.0 cm) using a linear gradient of TEAB (0 – 1.0 M) (0.020 mmol, 51% yield), followed by semi-preparative RP HPLC and subjected to enzymatic cleavage according to the general procedure.

2′,3′-cyclophospho N6-(8-azido-3,6-dioxyoctano-1-amino) adenosine 5′- phosphosulfate **11′**:
MS-ESI: $[M-H]^-$. calcd for $C_{16}H_{23}N_8O_{14}P_2S^-$, 645.1; found, 645.2.

3′-phospho N6-(8-azido-3,6-dioxyoctano-1-amino) adenosine 5′- phosphosulfate **11**:
MS-ESI: $[M-H]^-$. calcd for $C_{16}H_{25}N_8O_{15}P_2S^-$, 663.1; found, 662.8.

9. General Procedure for the Synthesis of 12,13

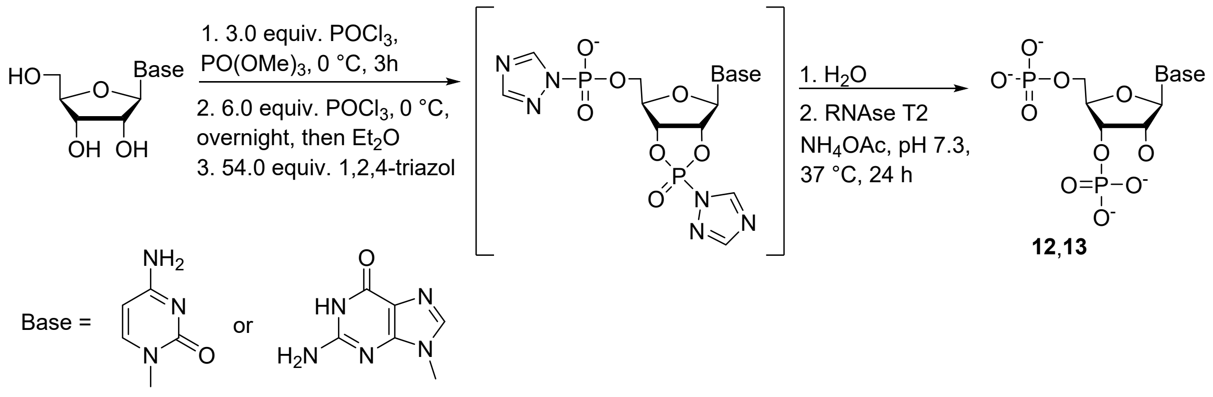

Scheme S6. Synthesis of compounds **12,13**.

To a solution of nucleoside (1.0 equiv.) in trimethyl phosphate (0.2 - 0.5 M), $POCl_3$ (3.0 equiv.) was added at 0 °C and stirred for 3 h. The progress of the reaction was analyzed by RP HPLC. When complete conversion to product was observed, a second portion of $POCl_3$ (6.0 equiv.) was added. The mixture was left overnight at 0 °C and allowed to warm up to 24 °C, then poured into cold diethyl ether and centrifuged. Supernatant was discarded and the precipitate was diluted in trimethyl phosphate (2 mL) followed by addition of 1,2,4-triazole (54.0 equiv.). After 1 h the reaction was quenched with 10 volumes of water and purified by DEAE-Sephadex using a linear gradient of TEAB (0 – 0.8 M), followed by semi-preparative RP-HPLC and subjected to enzymatic cleavage according to the general procedure.

a. 3’-Phosphocytidine 5’-diphosphate 12

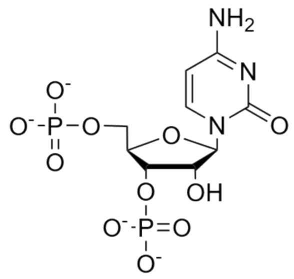

Following the general procedure, cytosine (50 mg, 0.21 mmol, 1.0 equiv.), $POCl_3$ (95 mg, 0.62 mmol, 3.0 equiv.) in trimethyl phosphate (1.0 mL) were used at 0 °C. After 3 h, the second portion of $POCl_3$ (190 mg, 1.24 mmol, 6.0 equiv.) was added. The mixture was left overnight at 0 °C, then poured into cold diethyl ether and centrifuged. Supernatant was discarded and the precipitate was diluted in trimethyl phosphate (2.0 mL) followed by addition of 1,2,4-triazole (770 mg, 11.3 mmol, 54.0 equiv.). After 1 h the reaction was quenched with 10 volumes of water and purified by DEAE-Sephadex A-25 column (4.0 × 15.0 cm) using a linear gradient of TEAB (0 – 1.0 M), to obtain the product **12′** as

triethylammonium salt (0.08 mmol, 39% yield), followed by semi-preparative RP-HPLC and subjected to enzymatic cleavage according to the general procedure.

2′,3′-cyclophospho cytidine 5′-diphosphate **12′**:

^{1}H NMR (500 MHz, D_2O): δ 7.80 (d, *J* = 7.6 Hz, 1H), 6.08 (d, *J* = 7.6 Hz, 1H), 6.01 (d, *J* = 3.0 Hz, 1H), 5.1 (ddd, *J* = 3.0, 6.7, 8.2 Hz, 1H), 5.07 – 5.00 (m, 1H), 4.53 – 4.49 (m, 1H), 4.22 – 4.07 (m, 2H).

^{31}P NMR (202 MHz, D_2O): δ 20.86 – 20.51 (m, 1P), 1.34 – 1.02 (m, 1P).

MS (ESI, m/z): $[M-H]^-$. calcd for $C_9H_{12}N_3O_{10}P_2^-$, 384.0; found, 384.0.

5′-diphosphate -3′-phosphocytidine **12**:

^{1}H NMR (500 MHz, D_2O): δ 8.01 (d, *J* = 7.5 Hz, 1H), 6.12 (d, *J* = 7.5 Hz, 1H), 6.02 (d, *J* = 4.6 Hz, 1H), 4.67 – 4.61 (m, 1H), 4.39 (t, *J* = 4.7 Hz, 2H), 4.19 – 4.07 (m, 2H), 3.81 – 3.73 (m, 1H).

^{31}P NMR (202 MHz, D_2O): δ 1.50 (d, *J* = 8.7 Hz, 1P), 1.04 (s, 1P).

HRMS: $[M-H]^-$. calcd for $C_9H_{14}N_3O_{11}P_2^-$, 402.01090; found, 402.01125.

b. 5’-Diphosphate -3’-phosphoguanosine 13

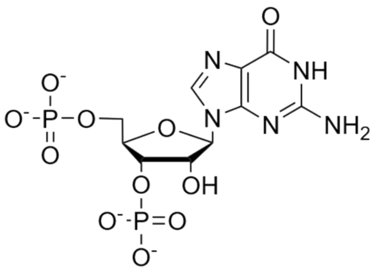

Following the general procedure, guanosine (50 mg, 0.18 mmol, 1.0 equiv.), $POCl_3$ (80 mg, 0.53 mmol, 3.0 equiv.) in trimethyl phosphate (1.0 mL) were used at 0 °C. After 3 h, the second portion of $POCl_3$ (160 mg, 1.06 mmol, 6.0 equiv.) was added. The mixture was left overnight at 0 °C, then poured into cold diethyl ether and centrifuged. Supernatant was discarded and the precipitate was diluted in trimethyl phosphate (2.0 mL) followed by addition of 1,2,4-triazole (660 mg, 9.54 mmol, 54.0 equiv.). After 1 h the reaction was quenched with 10 volumes of water and purified by DEAE-Sephadex A-25 column (4.0 × 15.0 cm) using a linear gradient of TEAB (0 – 1.0 M), to obtain the product **13′** as triethylammonium salt (0.07 mmol, 37% yield), followed by semi-preparative RP-HPLC and subjected to enzymatic cleavage according to the general procedure.

2’,3’-cyclophospho guanosine 5’-diphosphate **13′**:

^{1}H NMR (500 MHz, D$_2$O): δ 8.02 (s, 1H), 6.16 (d, *J* = 3.7 Hz, 1H), 5.42 (ddd, *J* = 3.7, 6.7, 10.1 Hz, 1H), 5.22 (ddd, *J* = 3.7, 6.7, 8.8 Hz, 1H), 4.59 – 4.53 (m, 1H), 4.21 – 4.09 (m, 2H).

^{31}P NMR (202 MHz, D$_2$O): δ 20.83 – 20.63 (m, 1P), 1.23 – 1.00 (m, 1P).

MS (ESI, m/z): [M-H]$^-$. calcd for $C_{10}H_{12}N_5O_{10}P_2^-$, 424.0; found, 423.9.

5’-diphosphate -3’-phosphoguanosine **13**:

^{1}H NMR (500 MHz, D$_2$O): δ 8.12 (s, 1H), 5.98 (d, *J* = 6.3 Hz, 1H), 4.88 – 4.81 (m, 2H), 4.57 – 4.50 (m, 1H), 4.18 – 4.09 (m, 2H), 3.81 – 3.74 (m, 2H).

^{31}P NMR (202 MHz, D$_2$O): δ 0.25 (s, 1P), 0.08 (d, *J* = 8.7 Hz, 1P).

HRMS: [M-H]$^-$. calcd for $C_{10}H_{14}N_5O_{11}P_2^-$, 442.01705; found, 442.01757.

10. Synthesis of 5’,3’-diphosphate 2’-fluoro adenosine 14

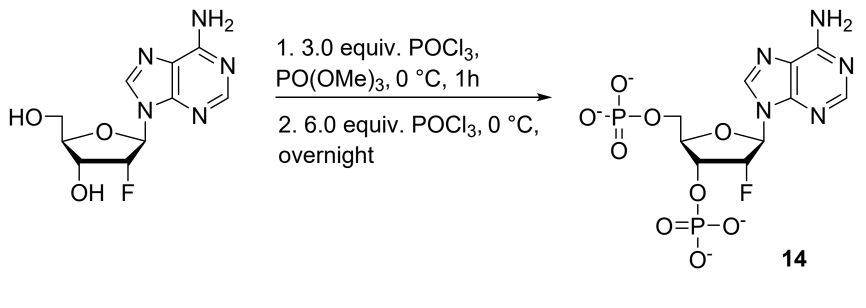

Scheme S7. Synthesis of 5’,3’-diphosphate 2’-fluoro adenosine **14**.

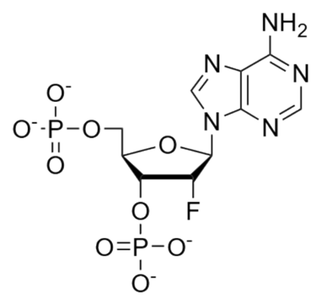

Following the general procedure, 2’-F-adenosine (52 mg, 0.20 mmol, 1.0 equiv.), $POCl_3$ (90 mg, 0.58 mmol, 3.0 equiv.) in trimethyl phosphate (1.0 mL) were used at 0 °C. After 1 h, the second portion of $POCl_3$ (180 mg, 1.17 mmol, 6.0 equiv.) was added. The mixture was left overnight at 0 °C, then the reaction was quenched with 10 volumes of water and purified by semi-preparative RP HPLC to obtain the target product **14** in 10% yield (8 mg, 0.02 mmol).

<u>5’,3’-diphosphate 2’-fluoro adenosine **14**:</u>

^{1}H NMR (500 MHz, D_2O): δ 8.48 (s, 1H), 8.27 (s, 1H), 6.47 (dd, *J* = 15.6, 2.5 Hz, 1H), 5.62 – 5.42 (m, 1H), 5.08 – 4.93 (m, 1H), 4.52 (d, *J* = 6.0 Hz, 1H), 4.35 – 4.11 (m, 2H).

^{31}P NMR (202 MHz, D_2O): δ 0.54 (s, 1P), 0.06 (d, *J* = 8.7 Hz, 1P).

^{19}F NMR (471 MHz, D_2O): δ -75.60 (s, 1F).

HRMS: $[M-H]^-$. calcd for $C_{10}H_{13}FN_5O_9P_2^-$, 428.01780; found, 428.01815.

11. Synthesis of 5’,3’-adenosine dithiophosphonate 15

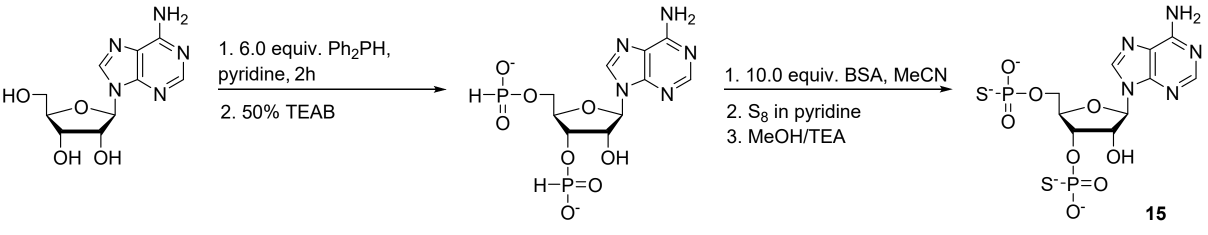

Scheme S8. Synthesis of 5’,3’-adenosine dithiophosphonate **15**.

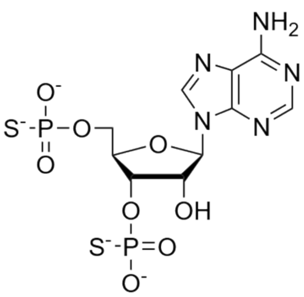

To a solution of adenosine (2.0 g, 7.49 mmol, 1.0 equiv.) in pyridine (37.0 ml), 1-diphenyl phosphonate (4.30 ml, 22.47 mmol, 6.0 equiv.) was added, and the reaction mixture was stirred for 2 h at room temperature, then the reaction was quenched with a 1:1 mixture of TEAB:H_2O and purified by DEAE-Sephadex A-25 column (6.0 × 16.0 cm) using a linear gradient of TEAB (0 – 0.8 M), to obtain 5’,3’,(2’)-H adenosine diphosphonate in the form of two isomers (1:0.5 3’-, 2’-) in 93% yield (6.97 mmol). Next, P_HAP_H (100 mg, 0.74 mmol, 1.0 equiv.), BSA (0.5 mL, 2.02 mmol, 10.0 equiv.) were mixed in acetonitrile (2.0 ml). After 1 h, S_8 suspended in pyridine was added to the mixture and the reaction mixture was stirred for 24 h at room temperature. Then it was

quenched with a mixture of MeOH/TEA and purified by DEAE-Sephadex A-25 column (6.0 × 16.0 cm) using a linear gradient of TEAB (0 – 1.2 M), to obtain 5’,3’-adenosine dithiophosphonate **15** in 13% yield (75 mg, 0.09 mmol).

<u>5’,3’,(2’)-H adenosine diphosphonate (P_HAP_H):</u>

HRMS: [M-H]$^-$. calcd for $C_{10}H_{14}N_5O_8P_2^-$, 394.03231; found, 394.03235.

<u>5’,3’-adenosine dithiophosphonate **15**:</u>

^{1}H NMR (500 MHz, D_2O): δ 8.67 (s, 1H), 8.28 (s, 1H), 6.19 (d, *J* = 6.4 Hz, 1H), 5.00 (d, *J* = 10.6, 5.0, 2.9 Hz, 1H), 4.90 – 4.85 (m, 1H), 4.71 – 4.64 (m, 1H), 4.25 – 4.14 (m, 2H).

^{31}P NMR (202 MHz, D_2O): δ 50.38 (bs, 1P), 49.45 (bs, 1P).

HRMS: [M-H]$^-$. calcd for $C_{10}H_{14}N_5O_8P_2S_2^-$, 457.97645; found, 457.97673.

12. Synthesis of 5’,3’-dithiophosphate-2-O-pentynyl adenosine 16

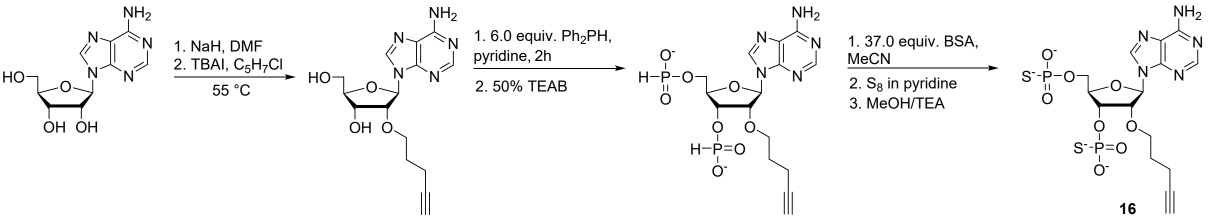

Scheme S9. Synthesis of 5’,3’-dithiophosphonate-2-O-pentynyl adenosine **16**.

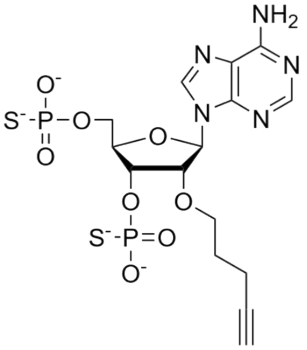

To a solution of adenosine (1.00 g, 3.74 mmol, 1.0 equiv.) in DMF (75.0 ml), NaH (0.60 g, 15.0 mmol, 4.0 equiv.) were used at 0 °C. After 1 h TBAI (0.30 g, 0.75 mmol, 0.2 equiv.) and 5-chloro-1-pentyne (1.07 g, 10.49 mmol, 2.8 equiv.) were added and the reaction mixture was stirred for 5 days. The product was purified by column chromatography (12% MeOH in DCM) to obtain 2-O-pentynyl adenosine in 18% yield (219 mg, 0.66 mmol).

Then, to a solution of 2-O-pentynyl adenosine (50 mg, 0.15 mmol, 1.0 equiv.) in pyridine (1.0 ml), diphenyl phosphonate (225 mg, 0.90 mmol, 6.0 equiv.) was added, and the reaction mixture was stirred for 4 days at room temperature, then the reaction was quenched with

a 1:1 mixture of TEAB:H_2O and purified by DEAE-Sephadex A-25 column (4.0 × 15.0 cm) using a linear gradient of TEAB (0 – 0.6 M), to obtain 5',3'-H- diphosphonate 2-O-pentynyl adenosine in 85% yield (0.12 mmol).

Next, 2-O-pentynyl P_HAP_H (56.0 mg, 0.08 mmol, 1.0 equiv.), BSA (0.56 g, 2.96 mmol, 37.0 equiv.) were mixed in acetonitrile (1.0 ml). After 1 h, S_8 suspended in pyridine was added to the mixture and the reaction mixture was stirred for 24 h at room temperature. Then it was quenched with a mixture of MeOH/TEA and purified by DEAE-Sephadex A-25 column (4.0 × 15.0 cm) using a linear gradient of TEAB (0 – 1.1 M) (50.3 mg, 0.06 mmol, 78% yield), followed by semi-preparative RP HPLC to obtain 5',3'-dithiophosphate-2-O-pentynyl adenosine **16** in 32% (23.0 mg, 0.02 mmol).

5',3'-H-diphosphonate-2-O-pentynyl adenosine (2-O-pentynyl P_HAP_H):

^{1}H NMR (500 MHz, D_2O): δ 8.52 (s, 1H), 8.29 (s, 1H), 7.58 (d, *J* = 84.8 Hz, 1H), 6.21 (d, *J* = 75.1 Hz, 1H), 6.22 (d, *J* = 7.3 Hz, 1H), 5.00 (ddd, *J* = 10.1, 5.1, 2.1 Hz, 1H), 4.68 (dd, *J* = 7.3, 4.6 Hz, 1H), 4.61 – 4.56 (m, 1H), 4.14 (qdd, *J* = 11.9, 6.4, 3.1 Hz, 2H), 3.82 (dt, *J* = 9.9, 5.6 Hz, 1H), 3.61 (ddd, *J* = 9.9, 7.8, 4.9 Hz, 1H), 3.12 – 3.03 (m, 1H), 2.13 – 2.02 (m, 3H).

^{31}P NMR (202 MHz, D_2O): δ 7.64 (dd, *J* = 650, 6.5 Hz, 1P), 6.48 (dd, *J* = 650.1, 9.9 Hz, 1P).

MS (ESI, m/z): $[M-H]^-$. calcd for $C_{15}H_{20}N_5O_8P_2^-$, 460.1; found, 460.1.

5',3'-2-O-pentynyl adenosine dithiophosphonate **16**:

^{1}H NMR (500 MHz, D_2O): δ 8.73 (s, 1H), 8.31 (s, 1H), 6.24 (d, *J* = 7.5 Hz, 1H), 5.11 (dd, *J* = 10.9, 5.0 Hz, 1H), 4.68 (t, *J* = 6.3 Hz, 1H), 4.48 – 4.40 (m, 1H), 4.29 – 4.17 (m, 2H), 3.93 (dd, *J* = 10.1, 5.5 Hz, 1H), 3.65 – 3.56 (m, 2H), 2.12 – 2.01 (m, 3H).

^{31}P NMR (202 MHz, D_2O): δ ^{31}P NMR (202 MHz, D_2O) δ 50.28 – 49.44 (m, 1P), 0.05 (s, 1P).

HRMS: $[M-H]^-$. calcd for $C_{15}H_{20}N_5O_8P_2S_2^-$, 524.02340; found, 524.02382.

13. General Procedure for the Synthesis of Fluorinated PAPS 17–19

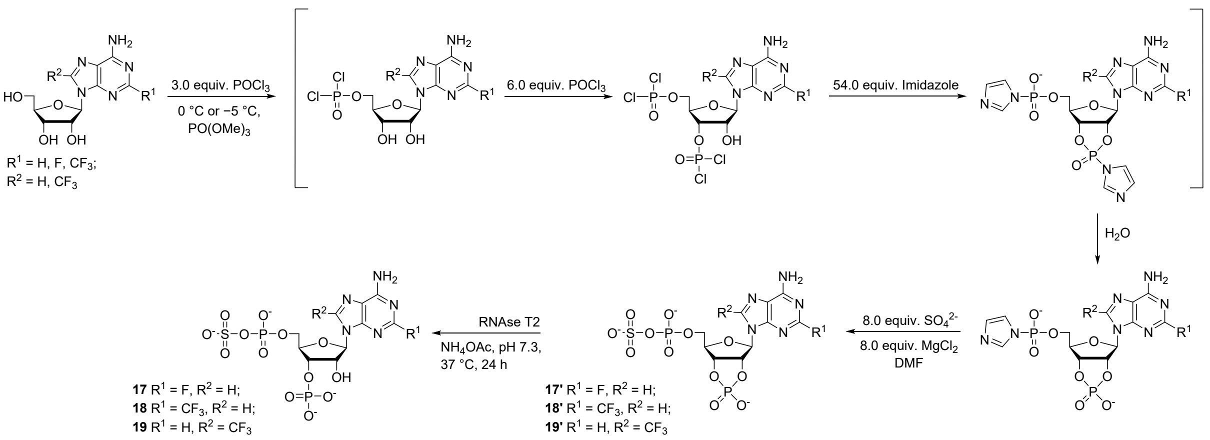

Scheme S10. Synthesis of fluorinated PAPS analogs **17–19**.

To a solution of nucleoside (1.0 equiv.) in trimethyl phosphate (0.2 - 0.5 M), $POCl_3$ (3.0 equiv.) was added at 0 °C and stirred for 3 h. The progress of the reaction was analyzed by RP HPLC. When complete conversion to product was observed, a second portion of $POCl_3$ (6.0 equiv.) was added. The mixture was left overnight at 0 °C and allowed to warm up to 24 °C, then poured into cold diethyl ether and centrifuged. Supernatant was discarded and the precipitate was diluted in trimethyl phosphate (2 mL) followed by addition of imidazole (54.0 equiv.). After 1 h the reaction was quenched with 10 volumes of water and purified by DEAE-Sephadex using a linear gradient of TEAB (0 – 0.8 M), to obtain the product as triethylammonium salt. The imidazole derivative should be stored at -20 °C.

2',3'-Cyclophosphoadenosine 5'-phosphorimidazolide derivative was diluted in DMF, and bis(tributylammonium) sulfate (8.0 equiv.) and anhydrous $MgCl_2$ (8.0 equiv.) were added. The reaction was stirred for at least 18 h (until total conversion of starting material was observed by RP-HPLC) and then quenched by addition of equimolar solution of EDTA in H_2O (the pH was adjusted to 7.0 by the addition of $NaHCO_3$).

Enzymatic cleavage of cyclic PAPS (10 mg/mL) was performed with RNAse T2 (750 U, 5 μL/10 mg, MoBiTec) in 50 mM ammonium acetate buffer, pH 7.3.[7] The reaction mixture was placed in thermoblock at 37 °C, 300 rpm. The reaction progress was monitored by RP HPLC. After 24 h the reaction was filtered through 10 K Amicon Ultra-

0.5 mL Centrifugal Filters. The concentrate was discarded and the flow-through was for NMR experiments (recovery greater than 90%).

a. 3'-Phospho-2-(fluoro) adenosine 5'-phosphosulfate 17

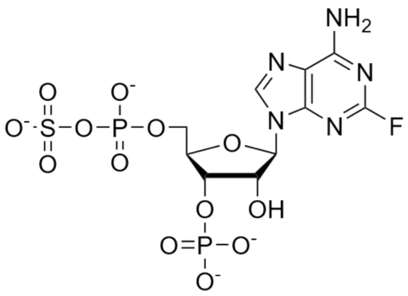

Following the general procedure, 2-fluoroadenosine (110 mg, 0.39 mmol, 1.0 equiv.), $POCl_3$ (177 mg, 1.16 mmol, 3.0 equiv.) in trimethyl phosphate (2.0 mL) were used at 0 °C. After 3 h, the second portion of $POCl_3$ (354 mg, 2.31 mmol, 6.0 equiv.) was added. The mixture was left overnight at 0 °C, then poured into cold diethyl ether and centrifuged. Supernatant was discarded and the precipitate was diluted in trimethyl phosphate (2.0 mL) followed by addition of imidazole (1414 mg, 20.8 mmol, 54.0 equiv.). After 1 h the reaction was quenched with 10 volumes of water and purified by DEAE-Sephadex A-25 column (6.0 × 16.0 cm) using a linear gradient of TEAB (0 – 0.8 M), to obtain the product as triethylammonium salt (0.11 mmol, 28% yield). Next, bis(tributylammonium) sulfate (204 mg, 0.43 mmol, 4.0 equiv.) and $MgCl_2$ (86 mg, 0.90 mmol, 8.0 equiv.) were added in DMF (2.0 mL). 2'3'-Cyclophospho-2-(fluoro) adenosine 5'-phosphate **17′** was purified by DEAE-Sephadex A-25 column (4.0 × 15.0 cm) (0.08 mmol, 72% yield), followed by semi-preparative RP HPLC to obtain cyclic 2-F-PAPS in 52% yield (28 mg, 0.41 mmol). Enzymatic cleavage was conducted according to the general procedure to get a stock solution of **17** for NMR experiments (recovery greater than 90%).

^{1}H NMR (500 MHz, D_2O) δ 8.49 (s, 1H), 6.05 (d, *J* = 6.9 Hz, 1H), 4.85 – 4.72 (m, overlap with D_2O, 2H), 4.60 – 4.57 (m, 1H), 4.29 – 4.20 (m, 2H).

^{31}P NMR (202 MHz, D_2O): δ 3.64 – 3.29 (m, 1P), -9.57 – -9.82 (m, 1P).

^{19}F NMR (471 MHz, D_2O): δ - 52.95 (s, 1F).

HRMS: $[M-H]^-$. calcd for $C_{10}H_{13}FN_5O_{13}P_2S^-$, 523.96953; found, 523.96991.

Analytical data for this compound was consistent with previously reported data.[2]

b. 3'-Phospho-2-(trifluoromethyl) adenosine 5'-phosphosulfate 18

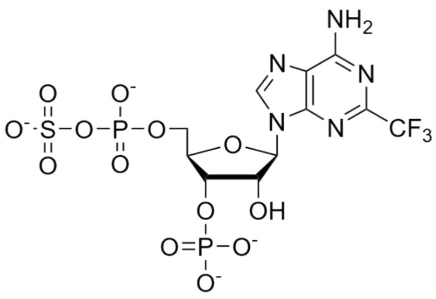

Following the general procedure 2-(trifluoromethyl)adenosine (49 mg, 0.15 mmol, 1.0 equiv.), 2.6-lutidine (67 mg, 0.45 mmol, 3.0 equiv.), $POCl_3$ (67 mg, 0.44 mmol, 3.0 equiv.) in trimethyl phosphate (1.0 mL) were used at -5 °C. After 4 h, the second portion of $POCl_3$ (134 mg, 0.87 mmol, 6.0 equiv.) was added. The mixture was left overnight at – 5 °C and then poured into cold diethyl ether and centrifuged. Supernatant was discarded and the precipitate was diluted in trimethyl phosphate (1.0 mL) followed by addition of imidazole (534 mg, 7.85 mmol, 54 equiv.). After 1 h reaction was quenched with 10 volumes of water and purified by DEAE-Sephadex A-25 column (4.0 × 15.0 cm) using a linear gradient of TEAB (0 – 0.8 M), to obtain the product as triethylammonium salt (0.02 mmol, 11% yield). Next, bis(tributylammonium) sulfate (17 mg, 0.04 mmol, 2.0 equiv.) and $MgCl_2$ (16 mg, 0.17 mmol, 11.0 equiv.) were added in DMF (0.5 mL). 2'3'-Cyclophospho-2-(trifluoromethyl) adenosine 5'-phosphate **18'** was purified by DEAE-Sephadex A-25 column (3.0 × 7.0 cm) using a linear gradient of TEAB (0 – 0.8 M) (0.01 mmol, 42% yield), followed by semi-preparative RP HPLC to obtain cyclic 2-CF_3-PAPS in 27% yield (1.2 mg, 0.02 mmol). Enzymatic cleavage was conducted according to the general procedure to get a stock solution of **18** for NMR experiments (recovery greater than 90%).

^{1}H NMR (500 MHz, D_2O): δ 8.68 (s, 1H), 6.27 (d, *J* = 6.7 Hz, 1H), 4.89 – 4.82 (m, 2H), 4.64 – 4.60 (m, 1H), 4.29 – 4.25 (m, 2H).

^{31}P NMR (202 MHz, D_2O): δ 1.87 – 1.16 (m, 1P), -9.61 – -10.17 (m, 1P).

^{19}F NMR (471 MHz, D_2O): δ - 69.24 (s, 3F).

HRMS: $[M-H]^-$. calcd for $C_{11}H_{13}F_3N_5O_{13}P_2S^-$,573.96634; found, 573.96687.

Analytical data for this compound was consistent with previously reported data.[2]

c. 3'-Phospho-8-(trifluoromethyl) adenosine 5'-phosphosulfate 19

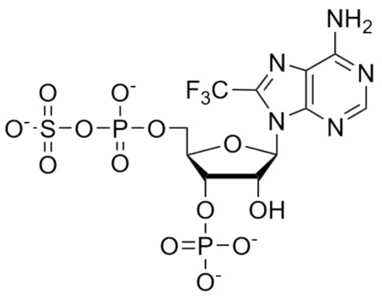

Following the general procedure 8-(trifluoromethyl)adenosine (101 mg, 0.30 mmol, 1.0 equiv.), 2.6-lutidine (96 mg, 0.90 mmol, 3.0 equiv.), $POCl_3$ (138 mg, 0.90 mmol, 3.0 equiv.) in trimethyl phosphate (2.0 mL) were used at 0 °C. After 3 h, the second portion of $POCl_3$ (276 mg, 1.8 mmol, 6.0 equiv.) was added. The mixture was left overnight at 0 °C and then poured into cold diethyl ether and centrifuged. Supernatant was discarded and the precipitate was diluted in trimethyl phosphate (2.0 mL) followed by addition of imidazole (1101 mg, 16.2 mmol, 54.0 equiv.). After 1 h reaction was quenched with 10 volumes of water and purified by DEAE-Sephadex A-25 column (6.0 × 16.0 cm) using a linear gradient of TEAB (0 – 0.8 M), to obtain the product as triethylammonium salt (0.04 mmol, 14% yield). Next, bis(tributylammonium) sulfate (83 mg, 0.18 mmol, 4.0 equiv.) and $MgCl_2$ (33 mg, 0.35 mmol, 8.0 equiv.) were added in DMF (2.0 mL). 2'3'-Cyclophospho-8-(trifluoromethyl) adenosine 5'-phosphate **19′** was purified by DEAE-Sephadex A-25 column (4.0 × 15.0 cm) using a linear gradient of TEAB (0 – 0.8 M) (0.02 mmol, 56% yield), followed by semi-preparative RP HPLC to obtain cyclic 8-CF_3-PAPS in 43% yield (7 mg, 0.01 mmol). Enzymatic cleavage was conducted according to the general procedure to get a stock solution of **19** for NMR experiments (recovery greater than 90%).

^{1}H NMR (500 MHz, D_2O): δ 8.38 (s, 1H), 6.13 (d, *J* = 6.1 Hz, 1H), 5.50 (t, *J* = 6.1 Hz, 1H), 5.01 – 4.95 (m, 1H), 4.56 – 4.51 (m, 1H), 4.42 – 4.28 (m, 2H).

^{31}P NMR (202 MHz, D_2O): δ 1.04 (d, *J* = 8.8 Hz, 1 P), -9.89 (t, *J* = 6.2 Hz, 12.5, 1P).

^{19}F NMR (471 MHz, D_2O): δ - 61.24 (s, 3F).

HRMS: $[M-H]^-$. calcd for $C_{11}H_{13}F_3N_5O_{13}P_2S^-$,573.96634; found, 573.96678.

Analytical data for this compound was consistent with previously reported data.[2]

14. HPLC assay

HPLC buffers: **A**: 0.1 M KH_2PO_4/ K_2HPO_4 buffer, pH 6;

B: buffer A: methanol 1:1.

The experiments were analyzed using Supelcosil LC-18-T column (4.6 × 250 mm, 5 μM, flow rate 1.3 mL min^{-1}) with linear gradient from buffer A to 50% of buffer B in 15 min.

15. Microscale thermophoresis assay

a. MST-based assay development

The measurements were performed using Monolith NT (Nanotemper).[9] The AtSOT18 and SULT1A3 proteins were labelled according to the following procedure. The RED-tris-NTA Dye (cat. no. MO-L008) was dissolved in 50 μl of 1-PBS-T buffer (0.05% Tween 20, pH 7.4) to obtain 5 μM dye solution. Then, 2 μl was taken and supplemented with 98 μl of 50 mM HEPES buffer, pH 6.5, 100 mM KCl, 0.05% Tween-20 to obtain 100 nM dye solution. The protein solution was prepared by diluting AtSOT18 in buffer to obtain 100 μl of protein at a concentration of 200 nM. Then, 90 μl of protein (200 nM) and 90 μl of dye (100 nM) were mixed and incubated at room temperature for 30 min. After this time, the mixture was centrifuged (10 min, 4°C, 14.000 rpm) and used in MST experiments. SULT1A3 labelling was performed in the same way.

To find optimal experimental conditions for AtSOT18 different binding buffers with varying pH and ionic strength using PAP as a model ligand were used. For each experiment, in a 0.2 ml tube, 10 μl of corresponding buffer (Figure S2, conditions **A**–**D**) was added. Then, 20 μl of PAP ligand (0.5-2 mM) was placed to the first test tube and the two-fold dilution method was used. A solution of labelled protein (10 μl) was added to tubes containing 10 μl of PAP ligand solution at appropriate concentrations and mixed. The solutions were filled into capillaries and measurements were performed after placing them in the apparatus. The obtained thermophoresis curves were analyzed using PALMIST92 software.[6] The highest stability of AtSOT18 was found in 50 mM HEPES, pH 6.5, 100 mM KCl, and 0.05% Tween-20 buffer, confirmed by determining the so-called inflection temperature (Ti) of the protein using differential scanning fluorimetry (DSF) (Figure S3). This method utilizes the spectroscopic properties of tryptophans present in the protein, which exhibit different emission maximums depending on the environment.

Interestingly, additional experiments demonstrated that ligand binding led to protein stabilisation (Figure S4).

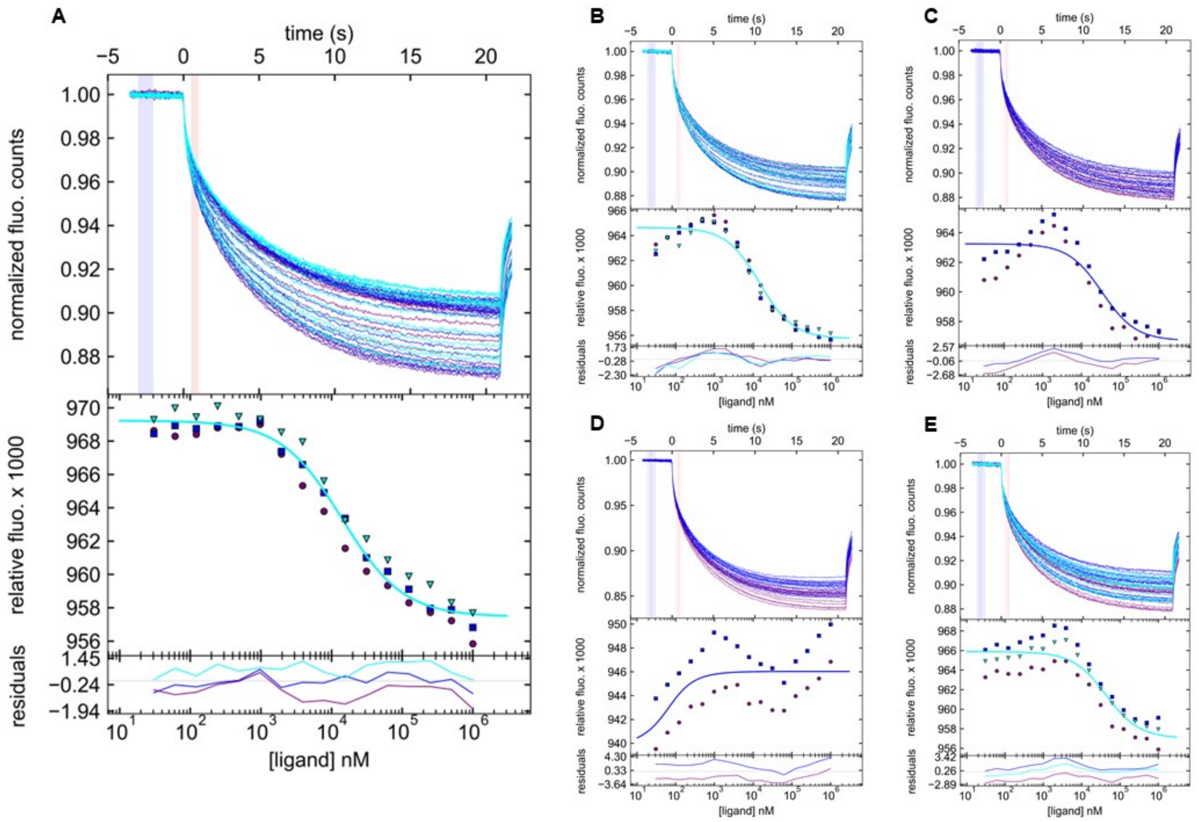

Figure S2. An MST binding curve of PAP for AtSOT18 under optimized conditions. **A**. 50 mM HEPES buffer, pH 6.5, 100 mM KCl, 0.05% Tween-20, conditions selected for further experiments; **B**. 50 mM HEPES, pH 7.5, 100 mM KCl, 0.05% Tween-20; **C**. 50 mM HEPES, pH 8.0, 100 mM KCl, 0.05% Tween-20; **D**. 50 mM HEPES, pH 8.5, 100 mM KCl, 0.05% Tween-20; **E**. 50 mM HEPES, pH 9.0, 100 mM KCl, 0.05% Tween-20.

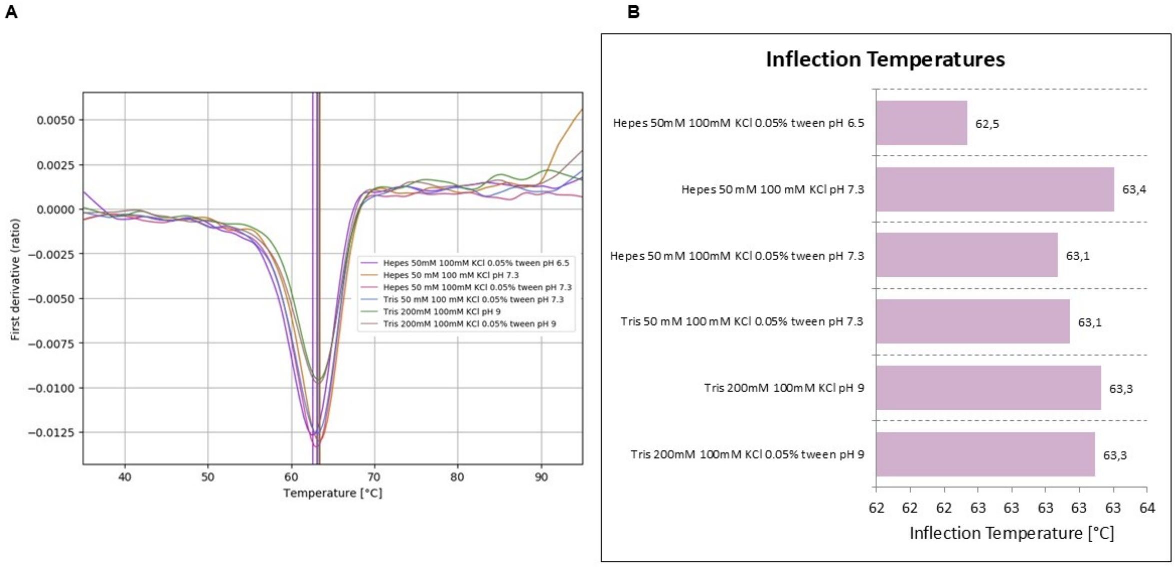

Figure S3. AtSOT18 protein stability results in selected buffers determined by the DSF method; **A**. Graph showing the dependence of the first derivative of the 350/330 nm ratio on temperature. **B**. Bar chart showing Ti values in selected buffers.

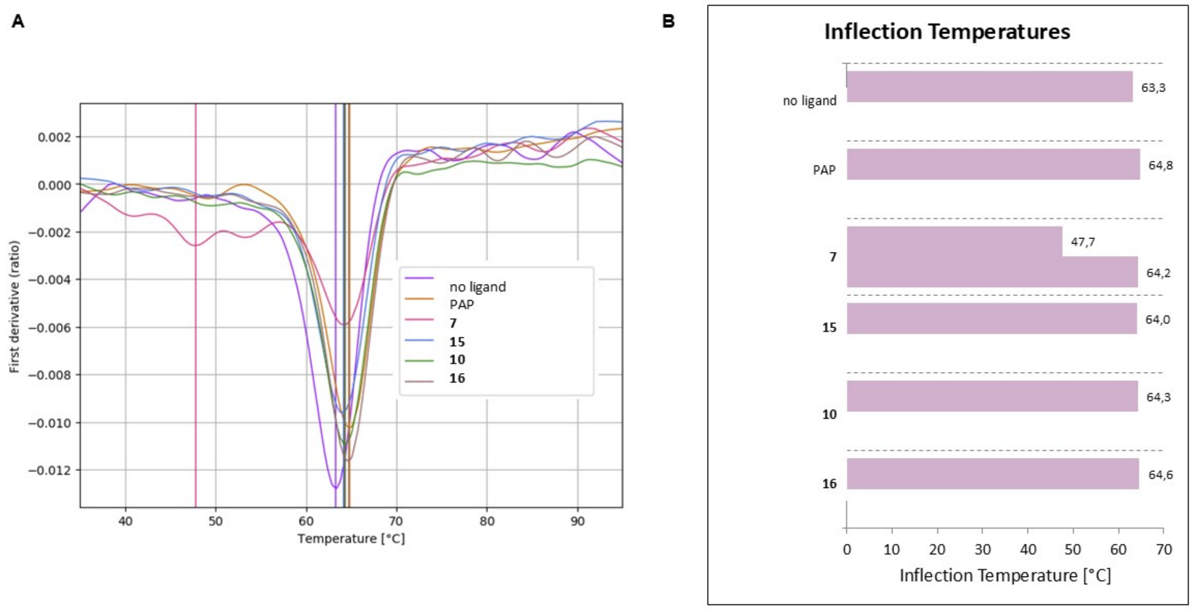

Figure S4. AtSOT18 protein stability results with selected ligands determined by the DSF method; **A**. Graph showing the dependence of the first derivative of the 350/330 nm ratio on temperature. **B**. Bar chart showing Ti values in selected buffers.

To find optimal experimental conditions for human sulfotransferase SULT1A3 similar experiments to AtSOT18 were conducted. First, the stability of the enzyme was determined by DSF measurements. Although the protein showed the highest Ti in HEPES buffer, pH 7.5, the graph of the first derivative of the 350/330 nm ratio versus temperature shows an inflection point at approximately 50°C (Figure S5). Thus, PBS-T buffer showing the highest protein stability was chosen for labelling and measurements. At this stage, it is worth mentioning that additional information on protein stability during the experiment can also be provided by the thermoelectric curve. Since the SULT1A3 enzyme is much more sensitive to minor changes in experimental conditions, the occurrence of numerous aggregates was observed.

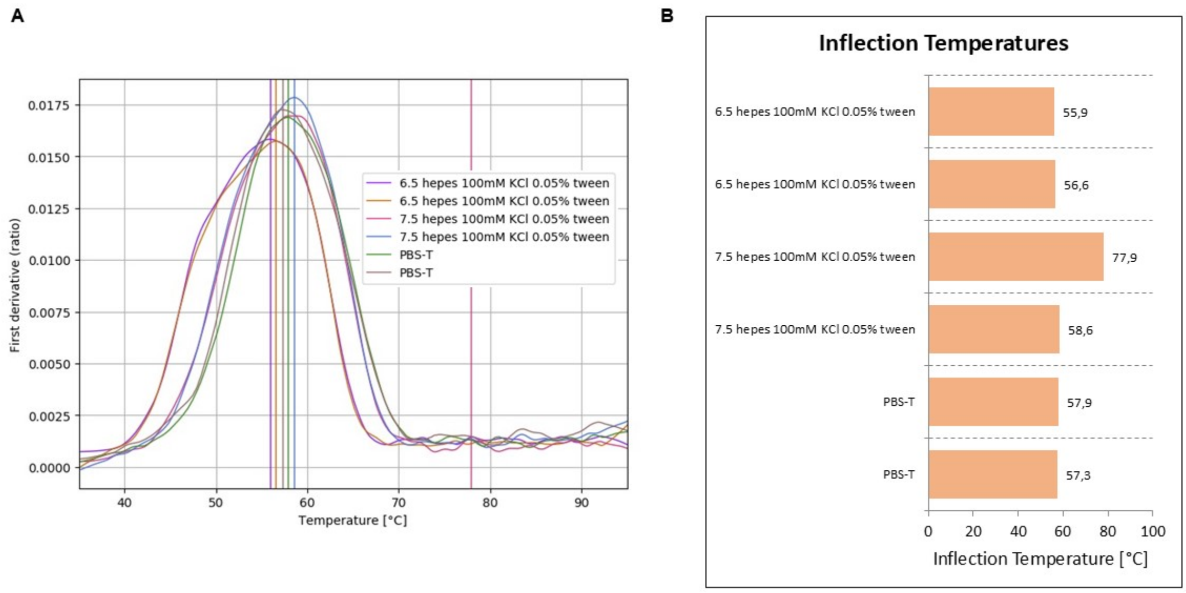

Figure S5. SULT1A3 protein stability results in selected buffers determined by the DSF method. **A**. Graph showing the dependence of the first derivative of the 350/330 nm ratio on temperature. **B**. Bar chart showing Ti values in selected buffers.

b. Evaluation of the compound library by an MST-based assay

Following previously optimized conditions, 16 tubes with a capacity of 0.2 ml were prepared. In 15 of them (except №1), 10 μl of buffer (for AtSOT18: 50 mM HEPES, pH 6.5, 100 mM KCl, 0.05% Tween-20; for SULT1A3: PBS-T buffer, pH 7.4, 0.05% Tween-20) was added. Then, 20 μl of ligand (0.5-2 mM) was added to the first test tube and the two-fold dilution method was used. A solution of labelled protein (10 μl) was added to 16 tubes containing 10 μl of ligand solution at appropriate concentrations and mixed. The solutions were filled into capillaries and measured as above-mentioned. To verify the reproducibility of the MST method under the previously optimized conditions, PAP was tested as a reference before each new measurement series. The data obtained for both AtSOT18 and SULT1A3 are presented in Table S1.

Table S1. Dissociation constant K_D values for AtSOT18 and SULT1A3 proteins with various ligands determined by MST-based assay.

	**Ligand**	**AtSOT18 K_D [μM][a]**	**n**	**SULT1A3 K_D [μM][b]**	**n**
1	AMP	1900 ± 1300	2	200 ± 400	2
2	ADP	1000 ± 500	3	13 ± 14	2
3	ATP	68 ± 13	3	n.d.	-
4	PAP	19 ± 3	3	0.7 ± 0.2	6
5[c]	PAP	0.5 ± 0.12	4	n.d.	-
6	cPAP	130 ± 80	2	n.d.	-
7	PAPS	13 ± 2	3	30 ± 16	3
8	**1**	12 ± 2	3	3 ± 2	2
9[c]	**1**	2.4 ± 0.9	3	n.d.	-
10	**2**	7.8 ± 1.2	3	28 ± 12	2
11	**3**	46 ± 2	2	14 ± 11	3
12[c]	**3**	23 ± 8	3	n.d.	-
13	**4**	90 ± 40	2	70 ± 20	2
14	**5**	180 ± 60	2	n.d.	-
15	**6**	280 ± 140	2	40 ± 30	3
16	**7**	16 ± 2	3	n.d.	-
17	**8**	20 ± 6	2	70 ± 40	3
18	**9**	8.4 ± 1.6	2	n.d.	-
19	**10**	22 ± 9	4	n.d.	-
20	**11**	93 ± 10	4	390 ± 150	3
21	**12**	2200 ± 500	3	n.d.	-
22	**13**	670 ± 150	3	n.d.	-
23	**14**	17 ± 3	3	1000 ± 1.1	2
24	**15**	21 ± 8	2	n.d.	-
25	**16**	38 ± 10	3	20 ± 5	3
26[c]	**16**	29 ± 13	2	n.d.	-
27	**17**	43 ± 10	3	n.d.	-
28	**18**	390 ± 90	3	n.d.	-
29	**19**	130 ± 31	3	n.d.	-

[a] Conditions for AtSOT18: 50 mM HEPES, pH 6.5, 100 mM KCl, 0.05%; [b] Conditions for SULT1A3: PBS-T buffer; [c] Experiment was carried out with the addition of desulfosinigrin. n = number of repetitions; n.d. = no data.

16. ^{19}F NMR-Monitored Sulfotransferase Experiments Assay

a. Screening with 2-CF_3-PAPS for AtSOT18

Enzymatic reactions were performed in 96-wells-F plates (Avantor 734-2327 VWR Tissue Culture Plate 96-Wells-F, 0,075 – 0.20 ml, U-Bottom). Each well contained 200 μM 2-CF_3-PAPS **18**, 100 μM desulfosinigrin, the 35 μM tested inhibitor candidate (0.96 μL of the 10 mM stock solution), and 100 nM protein in 83 mM TRIS buffer, pH 9.0, 9.2 mM $MgCl_2$, with addition of 10% D_2O (275 μL in total). The control reaction without the inhibitor (performed in triplicate) included 35 μM (0.96 μL) DMSO instead of the inhibitor. The control reaction without protein (performed in triplicate) included water instead of the enzyme. The reactions were placed in a thermoblock at 37 °C, 300 rpm for 40 min. After this time, each reaction was quenched by adding acetonitrile (275 μL) followed by 10 μL of buffered EDTA solution (20 mg mL^{-1} EDTA, 10 mg/mL^{-1} $NaHCO_3$) and was centrifuged, transferred to NMR tubes, and analyzed by ^{19}F NMR.

b. Screening with 8-CF_3-PAPS for SULT1A3

Enzymatic reactions were performed in 96-wells-F plates (Avantor 734-2327 VWR Tissue Culture Plate 96-Wells-F, 0,075 – 0.20 ml, U-Bottom). Each well contained 200 μM 8-CF_3-PAPS **19**, 200 μM dopamine, the 35 μM tested inhibitor candidate (0.96 μL of the 10 mM stock solution), and 100 nM protein in 6.7 mM K_2HPO_4, pH 7.4, with addition of 10% D_2O (275 μL in total). The control reaction without the inhibitor (performed in triplicate) included 35 μM (0.96 μL) DMSO instead of the inhibitor. The control reaction without protein (performed in triplicate) included water instead of the enzyme. The reactions were placed in a thermoblock at 37 °C, 300 rpm for 40 min. After this time, each reaction was quenched by adding acetonitrile (275 μL) followed by 10 μL of buffered EDTA solution (20 mg mL^{-1} EDTA, 10 mg/mL^{-1} $NaHCO_3$) and was centrifuged, transferred to NMR tubes, and analyzed by ^{19}F NMR.

References

1. M. Chrominski, M. Baranowski, S. Chmielinski, J. Kowalska and J. Jemielity, JOURNAL OF ORGANIC CHEMISTRY, 2020, 85, 3440-3453.
2. A. Mlynarska-Cieslak, M. Chrominski, T. Spiewla, M. Baranowski, M. Bednarczyk, J. Jemielity and J. Kowalska, ACS CHEMICAL BIOLOGY, 2022, 17, 661-669.
3. P. Wanat, S. Walczak, B. Wojtczak, M. Nowakowska, J. Jemielity and J. Kowalska, ORGANIC LETTERS, 2015, 17, 3062-3065.
4. S. Walczak, A. Nowicka, D. Kubacka, K. Fac, P. Wanat, S. Mroczek, J. Kowalska and J. Jemielity, CHEMICAL SCIENCE, 2017, 8, 260-267.
5. A. Jawalekar, N. Meeuwenoord, J. Cremers, H. Overkleeft, G. van der Marel, F. Rutjes and F. van Delft, JOURNAL OF ORGANIC CHEMISTRY, 2008, 73, 287-290.
6. T. Scheuermann, S. Padrick, K. Gardner and C. Brautigam, ANALYTICAL BIOCHEMISTRY, 2016, 496, 79-93.
7. J. HORWITZ, R. MISRA, J. ROZHIN, S. HELMER, A. BHUTA and S. BROOKS, BIOCHIMICA ET BIOPHYSICA ACTA, 1980, 613, 85-94.
8. M. Kalek, J. Jemielity, J. Stepinski, R. Stolarski and E. Darzynkiewicz, TETRAHEDRON LETTERS, 2005, 46, 2417-2421.
9. Z. Tibbs, K. Rohn-Glowacki, F. Crittenden, A. Guidry and C. Falany, DRUG METABOLISM AND PHARMACOKINETICS, 2015, 30, 3-20.
